# Physical Health in Patients with Post-COVID-19 6 and 12 Months after an Inpatient Rehabilitation: An Observational Study

**DOI:** 10.3390/jcm13133988

**Published:** 2024-07-08

**Authors:** Katrin Müller, Marcel Ottiger, Iris Poppele, Alois Wastlhuber, Michael Stegbauer, Torsten Schlesinger

**Affiliations:** 1 Institute of Human Movement Science and Health, Faculty of Behavioral and Social Sciences, Chemnitz University of Technology, 09107 Chemnitz, Germany; marcel.ottiger@hsw.tu-chemnitz.de (M.O.); iris.poppele@hsw.tu-chemnitz.de (I.P.); torsten.schlesinger@hsw.tu-chemnitz.de (T.S.); 2 BG Hospital for Occupational Disease Bad Reichenhall, 83435 Bad Reichenhall, Germany; alois.wastlhuber@bgklinik-badreichenhall.de (A.W.); michael.stegbauer@bgklinik-badreichenhall.de (M.S.)

**Keywords:** work-related COVID-19, post-COVID, physical health, rehabilitation, long-term outcomes

## Abstract

**Background:** Rehabilitation is an effective and feasible approach for post-COVID patients to improve physical health. However, knowledge regarding the long-term impact of rehabilitation on the physical health of these patients is lacking. **Methods:** Changes in physical health of 127 patients with COVID-19 as an occupational disease or work accident were assessed in a longitudinal observational study. Post-COVID symptoms, functional status, functional exercise capacity, endurance capacity, physical performance, quadricep strength, handgrip strength, motor balance ability, and self-reported physical performance were examined at the beginning as well as 6 and 12 months after the rehabilitation. Group differences concerning sex, age, acute COVID status, comorbidities prior to COVID-19, and aftercare interventions were also analysed. **Results:** Even 12 months after rehabilitation, the prevalence of post-COVID symptoms (28.6–94.7%) remained remarkably high in the study population. Significant improvements in various aspects of physical health were observed 6 (r = 0.288–0.755) and 12 months (r = 0.189–0.681) after the rehabilitation. Participants demonstrated enhanced endurance, strength, and balance function, as well as improvement in subjective physical ability. Significant group differences were observed between younger and older patients, those with mild–moderate and severe–critical COVID-19, and patients with and without pre-existing cardiovascular disease, metabolic disease, psychological disease, neuro-sensory disease, musculoskeletal disease, and exercising in an outpatient group. **Conclusions:** The study identifies persistent challenges in COVID-19 recovery, despite significant improvements in physical health 6 and 12 months after rehabilitation. Further research and the implementation of standardised approaches are required to enhance the outcomes of post-COVID rehabilitation, with a focus on developing personalised care strategies for long-term recovery.

## 1. Introduction

The COVID-19 pandemic, initially characterized as a respiratory illness, has revealed its multifaceted and complex nature by impacting various physiological systems. Consequently, it generates a wide spectrum of sequelae that profoundly impact affected individuals in the short, medium, and long term [1]. Workplaces, particularly healthcare settings, are high-risk settings for SARS-CoV-2 transmission due to intensive interpersonal and physical contacts with colleagues, clients, or patients [2,3]. A systematic review [4] revealed a higher prevalence of SARS-CoV-2 in healthcare professionals compared to the general population. In Germany, COVID-19 can be recognized as an occupational disease or accident if patients were affected by because of the insured person’s occupation, which is covered by insurance according to the German Social Insurance Code (§9 SGB VII) [5]. In Germany until March 2024, 357.396 cases of COVID-19 were recognized as occupational diseases (BK) with BK-No. 3101. In addition, 26.958 cases of COVID-19 were recognized as work-related accidents (according to the German Social Accident Insurance) [6].

Persistent manifestations and symptoms following an acute SARS-CoV-2 infection that cannot be attributed to an alternative diagnosis are classified as long COVID (lasting four weeks or longer) or post-COVID (from 12 weeks after the infection) [7]. Abundant evidence exists regarding the potential pathomechanisms involved in the development of post-COVID, including immune dysregulation, persistent organ damage, virus persistence, autoimmunity, endothelial dysfunction, dysfunctional neurological signalling and ongoing inflammatory processes [8,9]. Risk factors have been identified in various studies and include female sex, high BMI, older age, smoking, presence of comorbidities, severity of COVID-19 during the acute phase, previous hospitalization or ICU admission, and lack of vaccination [10,11,12]. The estimated prevalence of persistent post-COVID-19 symptoms in population-based cohort studies is 6–10%, with variability depending on factors such as virus variants, study population characteristics, and study design [8,13,14]. Post-COVID symptoms can be heterogeneous in number, quality, and duration. A pooled analysis of data from 22 countries identified three primary post-COVID symptom clusters: persistent fatigue, cognitive issues, and ongoing respiratory problems [14]. Among the most common post-COVID symptoms are effort intolerance, fatigue, sleep disorder, and dyspnoea 6–9 months after the acute infection [15].

COVID-19 patients commonly experience a range of physical impairments and symptoms that have lasting effects on their quality of life and functionality in daily living. These effects extend beyond the acute phase and encompass issues like impaired physical function and reduced activity levels [16]. Regardless of hospitalization status, studies revealed that physical impairments, functional limitations in activities of daily activities, and a decrease in health-related quality of life can persist after acute COVID-19 for several months (4–24 months). After acute SARS-CoV-2 infection with increased risk associated with age and hospitalization duration [15,17,18,19,20]. Additionally, physical impairments of post-COVID patients are linked to cognitive dysfunction [21] as well as fatigue [22].

Rehabilitation can play a crucial role in the recovery process, particularly regarding improvement of muscle weakness, dyspnoea, exercise intolerance, and quality of life after a SARS-CoV-2-infection [23]. According to (inter-) national guidelines [24,25], specific post-COVID rehabilitation programs are recommended to aid in the maintenance and restoration of biopsychosocial health. Respiratory rehabilitation has been found to enhance lung function, exercise capacity, and overall quality of life in post-COVID patients [26]. A multidisciplinary rehabilitation approach has similarly demonstrated positive effects on body composition, alleviating dyspnoea and fatigue, and enhancing physical capacity in post-COVID patients [27]. A recent systematic review of 16 studies involving over 1.000 adults also showed that pulmonary rehabilitation could improve dyspnoea, physical function, quality of life, anxiety, and depression [28]. Results by Ahmed et al. [29], Nopp et al. [30], and Pouliopoulou et al. [31] supported this and found improved exercise capacity in patients with post-COVID after pulmonary rehabilitation.

While there is an increasing body of knowledge regarding the symptomatology of post-COVID, there are still knowledge gaps regarding the long-term effects, especially over a period of more than 12 months post-infection. Rehabilitation and exercise therapy are crucial components of the recovery process for post-COVID patients, offering a multifaceted approach for improving physical function, quality of life, and overall health. Although short- and medium-term effects of rehabilitative measures have been documented in some studies [32], it is unclear whether these effects persist in the long-term. To bridge this gap, the current study examines the physical health of patients who acquired COVID-19 in the workplace 6 and 12 months after rehabilitation. Additionally, the study seeks to analyse group differences in physical health regarding sex, age, acute COVID status, comorbidities, and participation in aftercare interventions to identify potential associations.

## 2. Materials and Methods

This study (registered in the German Clinical Trials Register under DRKS00022928) was carried out in collaboration between Chemnitz University of Technology, Germany, and BG Hospital Bad Reichenhall, Germany. Ethical approval was obtained from the Ethics Committee of the Bavarian State Medical Association (approval number 21092) and the Ethics Committee of Chemnitz University of Technology (TU Chemnitz, Chemnitz, Germany), Faculty of Behavioural and Social Sciences (approval number V-427-17-KM-COVID-19-18022021). The comprehensive study protocol was previously published [33]. Here, we provide only the relevant information related to the current research question.

### 2.1. Study Design and Participants

In this prospective, longitudinal observational study, patients were enrolled by a study nurse following registration for rehabilitation at the German BG Hospital Bad Reichenhall, which specialises in occupational diseases, e.g., pulmonary diseases or post-traumatic stress disorders. Only patients in the post-acute phase of COVID-19 as recognized as an occupational disease or work-related accident were authorised for that inpatient rehabilitation after examination and approval by their respective accident insurance providers. Eligible patients gave their written consent. The study examines measurements taken at four different time points during the clinical stay as well as at home: the beginning (T1) and end (T2) of the inpatient rehabilitation period, as well as 6 months (T3) and 12 months (T4) after the end of rehabilitation. All participants underwent a multidisciplinary post-COVID rehabilitation program at BG Hospital, lasting on average 28.99 days (range: 21–42 days). This program included medical treatment and care, as well as physical and psychological interventions by specialized professionals. Müller et al. [33] and Table S1 in the supplementary file provided more detailed information on the rehabilitation program. Additionally, the results in physical health of the measurement time points T1 and T2 are published in Müller et al. [34].

At T1, 127 patients were enrolled. At T2, three participants were classified as dropped out: one due to discontinuation of rehabilitation and two due to loss of interest in study participation. At T3, three more participants dropped out, also due to lack of interest. At T4, six participants dropped out: two without giving a reason, one due to disinterest in participation, one after suffering a stroke shortly before T4, one due to being unable to complete the assessment at home or travel to the clinic in time, and one for work reasons. As a result of these dropouts, paired samples were analysed for 121 participants at T3 and 115 participants at T4. Considering missing values (reasons: e.g., missing questionnaire response; missing physical measures due to of health problems, e.g., knee pain) or missing clinical examination, the number of cases for each variable ranged from 95 to 117. Additionally, endurance capacity with spiroergometry was measured in a paired sample of 51 participants.

After the inpatient rehabilitation until T4 the participants received the following individual medically prescribed inpatient and outpatient post-COVID-19 treatments: 79% further medical treatment by the general practitioner, 23% repeated rehabilitation, 79% ambulatory physiotherapy, and 15% exercise in an outpatient group. The included patients were between 21 and 69 years of age (mean age = 50.62, standard deviation = 10.87). Among them, 85 patients worked in the healthcare sector (69 females), while 36 were classified as non-healthcare workers. In this study, 34% of patients had a medium SES and 65% had a high SES (one missing value), with none falling into the lowest SES category. At T1, 86% were overweight (BMI > 25 kg/m^2^). Detailed information about the study population characteristics can be found in Table 1.

### 2.2. Measurements of Sociodemographic Variables, Post-COVID Symptoms, Functional Status and after Care

Sociodemographic variables such as age, sex, socio-economic status, and education, were assessed via a questionnaire based on the German Health Interview and Examination Survey for Adults (DEGS) [35,36]. Socio-economic status (SES) ranged from 3.0 to 21.0 points and was categorized as low (3.0–7.7), medium (7.8–14.1), and high (14.2–21.0) [35]. The presence of post-COVID symptoms was documented using a questionnaire developed specifically for this study based on at the time valid guidelines [24] and differentiated into symptom clusters based on Bahmer et al. [37]. Additionally, a semi-standardized interview conducted by a physician during medical anamnesis evaluated pre-existing medical conditions. The Post-COVID-19 Functional Status (PCFS) scale assesses the impact of the disease on the functional status of patients, ranging from 0 (no limitations/symptoms in everyday life) to 4 (severe limitations/symptoms in everyday life) [38]. In addition, at time point T4, patients were asked by questionnaire to indicate post-COVID-19 treatments after inpatient rehabilitation (e.g., follow-up treatment by the general practitioner, ambulatory physiotherapy (e.g., breathing therapy), or exercising in an outpatient group).

### 2.3. Physical Performance Measurements

The six-minute walking test (6MWT) assessed the functional exercise capacity with a recommended minimal clinically important difference (MCID) of 30 m for determining significant improvements post-rehabilitation [39]. In addition, gait velocity was recorded for the first 10 metres of the six-minute walk. To assess participants’ endurance capacity, oxygen intake (VO2_max in L/Min_) and maximum power output (Watt_max_) were measured using a ramp protocol for spiroergometry (Vyntus CPX, Vyaire Medical, Höchberg, Germany). This measurement was taken from a small number of participants. The procedure was conducted with an ‘Ergoline’ ergometer (Bitz, Germany) and analysed using the software SentrySuite V3.20.3 (Vyaire Medical, Höchberg, Germany). Additionally, physical performance was examined with the 1-min sit-to-stand test (1MSTST; MCID ≥ 2 [40]. Quadricep strength was measured by isometric maximal strength test of quadriceps muscles, conducted with a functional press (Beinstemme V2 RD 836, Schnell Trainingsgeräte GmbH, Peutenhausen, Germany), and aktivSYSTEM software 7.1 (aktivKONZEPTE AG, St. Ingbert, Germany). A digital grip dynamometer (JAMAR^®^ Smart Hand Dynamometer, Performance Health Supply Inc., Cedarburg, WI, USA) was used to assess handgrip strength (HST) of the dominant hand. Motor balance ability was evaluated using the first and third part of the ‘Gleichgewichtstest-Rehabilitation’ (GGT-Reha, [41]), which comprises different tasks (six in each part) assessing both static as well as dynamic balance. The higher the total score (maximum of 36 points), the better the individual balance. Participants also self-reported their perceived physical performance status using a questionnaire comprising 10 items rated on a scale of 0–10 (0 = very bad, 10 = very well). The mean value of this scale is reported. Subjective post-COVID symptoms were also evaluated using a scale from 0 to 10 points, where 0 signifies the absence of symptoms and 10 represents severe symptomatology.

### 2.4. Statistical Analyses

SPSS software (version 29, SPSS Inc., Armonk, NY, USA) was used to analyse the data. Given the non-normal distribution of most parameters, the Wilcoxon signed-rank test compared variables across T1, T3, and T4. For the sake of completeness, the Friedman test was carried out, too. The results can be seen in Supplement Table S2 but will not be reported in the following sections. Group differences concerning sex (male vs. female), age (≤50 years vs. >50 years), acute COVID status (mild/moderate vs. severe/critical), comorbidities prior to COVID-19 (cardiovascular disease, respiratory disease, metabolic disease, psychological disease, neuro-sensory disease, musculoskeletal disease), and aftercare interventions (repeated rehabilitation, exercising in an outpatient group) were analysed using the Mann–Whitney U test. Only significant results regarding group differences at each measurement time point are presented in the text. Missing data were noted and are clearly presented in the tables, and *p*-values < 0.05 considered statistically significant. Effect sizes were reported as r, with an effect size of 0.1 representing a ‘small’ effect, 0.3 a ‘medium’ effect, and 0.5 a ‘large’ effect, following the guidelines of Fritz et al. [42].

## 3. Results

### 3.1. SARS-CoV-2 Infection and Post-COVID Symptoms

At the beginning of inpatient rehabilitation, the average duration since the infection with SARS-CoV-2 was 409.52 days (SD = 143.97, range: 124–813). Pneumonia was diagnosed in 30.0% of the infected individuals. According to the WHO classification for severity of COVID, 86 patients experienced a mild or moderate course of acute SARS-CoV-2 infection, while 35 patients had a severe or critical acute illness. Thirty-two participants were hospitalized for a duration ranging from 1 to 100 days, with nine of them receiving intensive care treatment for a duration of 5 to 21 days.

Before the infection with SARS-CoV-2, according to the patients’ reports, the following comorbidities were present: musculoskeletal disease (66%; e.g., arthrosis), metabolic disease (65%, e.g., type 2 diabetes), cardiovascular disease (48%, e.g., hypertension)), respiratory disease (43%, e.g., bronchial asthma), psychological disease (19%, e.g., depression), neurological disease (33%, e.g., migraine). Overall, 1.7% of the patients had no comorbidity prior to COVID-19.

**Figure 1:**
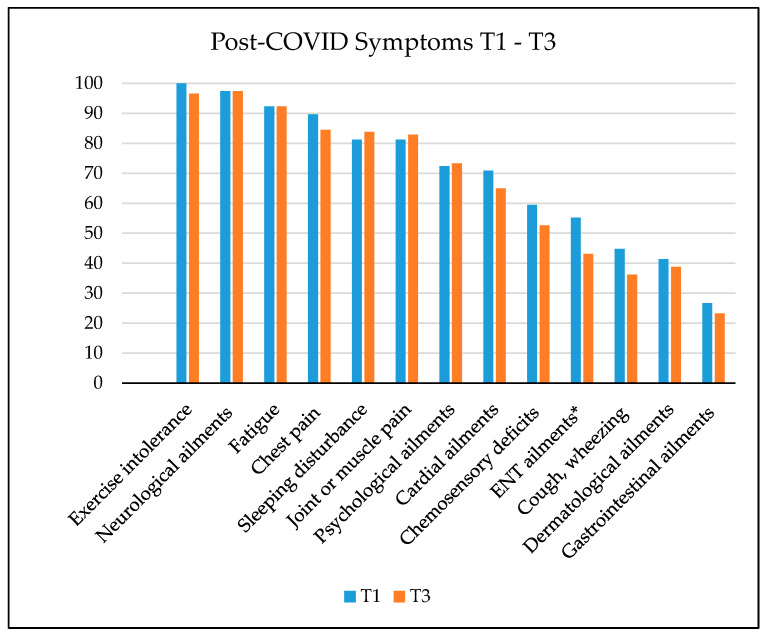
Symptom clusters of patients with post-COVID at T1 and T3 (paired samples). Significant differences are marked with * (*p* < 0.05). ENT—ear–nose–throat.

**Figure 2:**
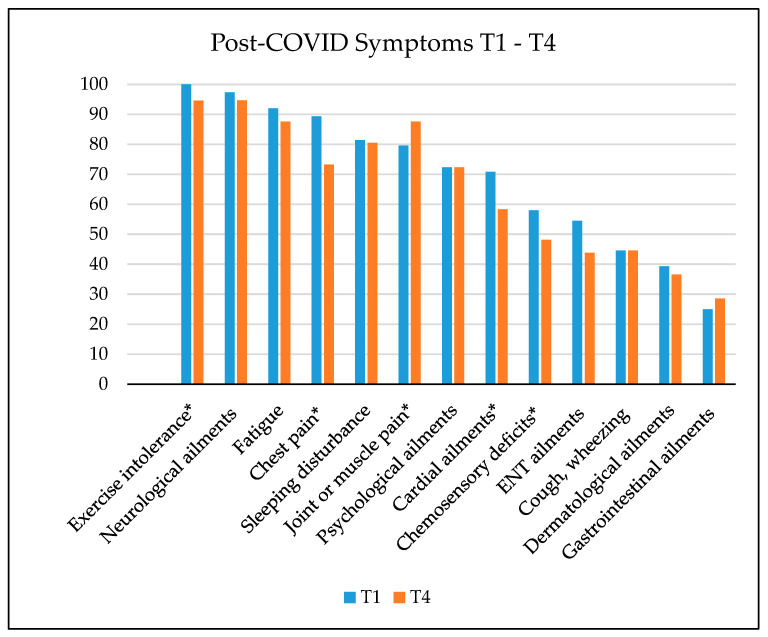
Symptom clusters of patients with post-COVID at T1 and T4 (paired samples). Significant differences are marked with * (*p* < 0.05). ENT—ear–nose–throat.

The percentage frequencies of subjectively reported post-COVID symptoms (compared to Bahmer et al. [37] at the three measurement time points are depicted in Figure 1 and Figure 2. The figure illustrates that most participants reported the following symptoms at T1: exertional intolerance, neurological complaints, and fatigue. Only the prevalence of ear–nose–throat ailments was significantly reduced at T3. The progression of post-COVID symptoms over time until T4 varies depending on the symptom severity. At T4 the prevalence of exercise intolerance, chest pain, cardiac ailments, and chemosensory deficits decreased significantly, whereas the prevalence of joint or muscle pain increased.

According to the PCFS score, the prevalence of no or negligible functional limitations was 9% and 2%, respectively. At T1, 36% of participants reported slight, 52% moderate, and 2% severe functional limitations. Compared to T1, PCFS score did not significantly change at T3 (*p* = 0.714; r = −0.036), whereas a significant improvement was observed at T4 (*p* = 0.022; r = −0.223); 12 months later at T4, 20% of patients exhibited no functional limitations, and 2% had negligible functional limitations. Most patients had slight (24%) or moderate (43%) functional limitations. Only 4% of participants reported severe functional limitations post-rehabilitation, and 7% of the patients did not answer.

### 3.2. Analysis of Physical Performance between T1 and T3

Table 2 presents the differences in physical health between T1 and T3. The analysis reveals significant improvements in various aspects of physical performance from T1 to T3. The 6MWT showed a significant increase in median distance covered, from 519.50 m at T1 to 588.00 m at T3 (*p* < 0.001). The gait velocity in the first 10 metres also decreased significantly from 1.48 m/s at T1 to 1.69 m/s at T3. Watt_max_ significantly differed between T1 (Mdn = 96.50 W) and T3 (Mdn = 108.00 W; *p* < 0.001). VO2_max_ showed a significant increase from 1.44 L/Min at T1 to 1.52 L/Min at T3 (*p* < 0.001). In the 1MSTST, a significant improvement (T1: Mdn = 20.00, T3: Mdn = 21.00, *p* = 0.003) was observed. Similarly, a significant increase (*p* < 0.001) was observed in quadricep strength, with a median of 97.78 kg at T1 and a median of 109.09 kg at T3. The change in handgrip strength from T1 (Mdn = 28.20 kg) to T3 (Mdn = 29.17 kg) was not significant. Balance function showed a significantly median increase (*p* = 0.002) from 25.00 points at T1 to 26.50 points at T3. Subjective physical health ratings increased from a median of 4.67 points at T1 to 5.11 points at T3 (*p* < 0.001). A significant change in subjective post-COVID symptoms was not recorded between the measurement points T1 and T3 (*p* > 0.05).

#### Group Differences between T1 and T3

Younger patients demonstrated a significantly greater increase in quadricep strength (Δ: Mdn = 15.33 kg) compared to older patients (Δ: Mdn = 7.71 kg; *p* = 0.037). A significant difference in subjective post-COVID symptoms was observed between patients with mild/moderate acute COVID-19 (Δ: Mdn = 1 point) and those with severe/critical COVID-19 (Δ: Mdn = −1 point; *p* = 0.009). Handgrip strength showed a significant difference between patients with and without pre-existing metabolic diseases. Patients with a pre-existing metabolic disease exhibited a decrease in handgrip strength (Δ: Mdn = −1.07 kg), while those without showed an increase (Δ: Mdn = 1.48 kg; *p* = 0.044). Additionally, significant differences in subjective post-COVID symptoms were found between patients with (Δ: Mdn = 1 point) and without pre-existing metabolic diseases (Δ: Mdn = 0 points; *p* = 0.034). Subjective physical ability differed significantly between patients with (Δ: Mdn = 0.22 points) and without pre-existing psychological diseases (Δ: Mdn = 1.22 points; *p* = 0.034). Moreover, a significant difference in handgrip strength was observed between patients with pre-existing neuro-sensory diseases and those without. Patients with neuro-sensory diseases demonstrated an increase in strength (Δ: Mdn = 1.51 kg), while patients without such diseases showed a decrease (Δ: Mdn = −1.38 kg; *p* = 0.029). Subjective post-COVID symptoms significantly differed between patients with pre-existing musculoskeletal diseases (Δ: Mdn = 1 point) and without pre-existing musculoskeletal diseases (Δ: Mdn = 0 points; *p* = 0.016). See Tables S3, S5, S7, S9, S11, S13, S15, S17, S19, and S21 for a comprehensive overview of the results and corresponding effect sizes.

### 3.3. Analysis of Physical Performance between T1 and T4

The analysis reveals significant improvements in various aspects of physical performance from the beginning of rehabilitation to 12 months after rehabilitation (see Table 3). The 6MWT showed a significant increase in median distance (*p* < 0.001) covered, from 521.00 m at T1 to 571.00 m at T4 (*p* < 0.001). Additionally, there was significant faster gait velocity at T4 (Mdn = 1.65 m/s) compared to T1 (Mdn = 1.47 m/s). Watt_max_ showed a significance increase between T1 (Mdn = 97.00 W) and T4 (Mdn = 108.00 W; *p* = 0.003). Similarly, there was a significant difference in VO2_max_ between the measurement points, with a median of 1.41 L/Min at T1 and 1.50 L/Min at T4 (*p* = 0.002). The number of repetitions in the 1MSTST was increased (T1: Mdn = 20.00; T4: Mdn = 21.00, *p* < 0.001) significantly with medium effect size. Significant increases were also observed in quadricep strength (T1: Mdn = 98.53 kg; T4: Mdn = 115.78 kg, *p* < 0.001), but not in handgrip strength (T1: Mdn = 27.47 kg; T4: Mdn = 28.92 kg). Significant improvements in balance function (T1: Mdn = 25.00 points; T4: Mdn = 26.50 points, *p* = 0.002) but with a small effect size were shown. Subjective physical ability ratings increased from a median of 4.78 points at T1 to 5.33 points at T4 (*p* < 0.001). There was no significant change in subjective post-COVID symptoms between the measurement points T1 and T4 (*p* > 0.05).

#### Group Differences between T1 and T4

Significant group differences were observed in quadricep strength from T1 to T4. Patients with a pre-existing cardiovascular disease showed a significantly greater increase in quadricep strength (Δ: Mdn = 28.66 kg) compared to those without the condition (Δ: Mdn = 13.05 kg; *p* = 0.003). Patients who underwent repeated rehabilitation showed a significantly greater increase in the number of repetitions of 1MSTST (Δ: Mdn = 4.00) compared to those who did not undergo repeated rehabilitation (Δ: Mdn = 2.00; *p* = 0.046). Patients exercising in an outpatient group experienced a significantly greater increase in Watt_max_ (Δ: Mdn = 28.00) compared to those who did not participate in outpatient group exercise (Δ: Mdn = 7.00; *p* = 0.004). Similarly, patients exercising in an outpatient group showed a significantly greater increase in VO2_max_ (Δ: Mdn = 0.31) compared to those not exercising in an outpatient group (Δ: Mdn = 0.08; *p* = 0.020). Patients exercising in an outpatient group also reported a significantly greater decrease in subjective post-COVID symptoms (Δ: Mdn = −1.00) compared to those not participating in outpatient group exercise (Δ: Mdn = 0.00; *p* = 0.036). Tables S4, S6, S8, S10, S12, S14, S16, S18, S20, and S22 provide a detailed overview of the results and effect sizes.

## 4. Discussion

A considerable proportion of individuals suffer from persistent symptoms and functional impairment long after acute COVID-19-infection. In this longitudinal observational study, we investigated the physical health of patients who acquired COVID-19 in the workplace, focusing on their rehabilitation journey (with mean duration of 29 days) and long-term outcomes.

Our study revealed that most participants reported exertional intolerance, neurological complaints, fatigue, chest pain, sleep disturbance, and joint or muscle pain at T1. Even 12 months after rehabilitation, prevalence rates remained remarkably high in the study population. It this context, it should be noted that the average duration of acute COVID-19 and the beginning of rehabilitation was 409 days. The persistent fatigue 12 months after rehabilitation is in line with systematic reviews on post-COVID, suggesting that prolonged fatigue is a frequent sequelae for many individuals recovering from acute COVID-19 [15,43]. In a comprehensive review including 70 studies with post-COVID patients of working age, fatigue, shortness of breath, muscle pain, and joint pain were the most reported symptoms [44]. In our study population, a higher prevalence of post-COVID symptoms was detected even 12 months after rehabilitation. Persistent symptoms can significantly impact the physical capacity and mental health of post-COVID patients, resulting in decreased work ability and increased sick leave [45,46]. Exercise intolerance is based on various underlying mechanisms. Aschman et al. [47] confirmed associations with capillary alterations (e.g., fewer capillaries, thicker capillary basement membranes) and immune dysregulations in skeletal muscles in post-COVID patients suffering from fatigue and post-exertional malaise. Furthermore, lower exercise capacity seems to be associated with changes in skeletal muscle metabolism and a transition to more fatigable fibers, which are rapidly fatigued [48].

Particularly concerning is the risk of chronification, especially in cases of post-infectious fatigue, a symptom affecting a significant proportion of post-COVID patients, as evidenced by long-term outcomes of other viral and non-viral infectious diseases (e.g., SARS virus, Q-fever, Lyme disease) [49]. Schaeffer et al. [50] found a relation between fatigue and reduced aerobic capacity, as measured by VO2peak during cardiopulmonary exercise testing. Fatigue remains a significant barrier to rehabilitation progress and warrants targeted interventions to address its underlying physiological and psychological contributors. This highlights the complex interplay between physical factors and fatigue in post-COVID recovery and underscores the importance of a holistic approach to post-COVID rehabilitation, addressing not only physical impairments but also psychological and cognitive as well as social sequelae. Especially considering that post-COVID is associated with an increased risk of developing a chronic fatigue syndrome or myalgic encephalomyelitis [8], it is essential to optimize rehabilitation contents and outcomes and promoting long-term well-being to provide appropriate, individualized pacing.

Findings from our study reveal significant improvements in various aspects of physical performance observed from the beginning of rehabilitation to different follow-up time points 6 and 12 month later. Participants demonstrated enhanced endurance, strength, balance, and overall physical function following a multidisciplinary post-COVID rehabilitation program. These improvements were evident in objective measures such as the 6MWT, the 1MSTST, quadricep strength, and balance. Performance on the 6MWT improved by an average of the minimal clinically important differences [39], particularly at T3 compared to T1, which was more than twice as high. The current result is comparable with the mean difference of 76.4 m reported in the study conducted by Kupferschmitt et al. [51]. Similarly, other studies have demonstrated significant improvements in exercise capacity and physical performance following rehabilitation programs. Nopp et al. [30] reported that the exercise capacity of post-COVID patients, measured by the 6MWT, improved significantly by an average of 62.9 m after a six-week pulmonary rehabilitation program, along with a 1-grade improvement on the PCFS scale. Additionally, an 8-week exercise program resulted in significant enhancements in physical performance and function, as evidenced by improvements in the 6MWT and 30-s STST among healthcare workers with post-COVID [52]. A recent systematic review confirms these significant positive effects after pulmonary rehabilitation on physical capacity (6MWT) [53]. The performance in the 6MWT declined slightly at T4 but remained significantly improved compared to T1. Therefore, it is important to maintain an active lifestyle to preserve these gains, for example, by taking part in outpatient exercise groups or using digital interventions at home [54]. Moreover, the endurance capacity measured by oxygen intake (VO2_max_) and maximum power output (Watt_max_) in our study, is comparable with previous research findings on post-COVID patients, indicating consistent benefits of rehabilitation on these measures. The recovery of the impaired exercise performance depends on an individualized physical exercise training taking into account the presence and severity of post-exertional malaise [32]. The effectiveness of rehabilitation interventions in improving physical performance aligns with previous research highlighting the role of structured interdisciplinary rehabilitation programs in post-COVID recovery. Studies have shown that rehabilitation can enhance cardiorespiratory fitness, muscle strength, exercise capacity, and physical function in individuals with post-COVID conditions [29,30,55]. It must be mentioned that studies examining physical performance objectively several months or years after post-COVID rehabilitation are rare. Therefore, future studies have to focus on understanding the long-term effects of post-COVID in physical performance and the underlying mechanisms as well the overall recovery.

In addition to the mentioned outcomes in the objectively measured physical health, there was also a subjective improvement in the perception of individual physical ability following rehabilitation as well as 6 and 12 months after the rehabilitation. Enhanced body awareness is essential for recognizing personal physical limits, particularly in the presence of fatigue and post-exertional malaise, and thus plays a pivotal role in the long-term maintenance of physical activity in daily life after rehabilitation. Evaluation of these individual limits can be achieved through a combination of subjective assessments, such as the Borg scale, and objective metrics, including heart and breath rate, facilitating the mitigation of the risk of overexertion.

Handgrip strength remained consistent over time without any notable changes. Other research also shows inconsistent results for changes in hand strength after rehabilitation [56,57]. The absence of a significant improvement in handgrip strength may be linked to the high prevalence of fatigue experienced by individuals, as muscle fatigue in handgrip is a component of overall fatigability. Studies have shown that there is a correlation between fatigue levels and handgrip strength [58,59], suggesting that the persistent fatigue observed in our study may have hindered improvements in handgrip strength. Do Amaral et al. [60] showed that dynapenia, characterized by reduced hand-grip strength (<30 Kgf (kilogram–force) in men and <20 Kgf in women), serves as a potential indicator of functional impairment in patients with post-COVID and is associated with lower muscle mass, poorer respiratory function, and worse 6MWT.

The role of physical and rehabilitation medicine physicians in developing individualized rehabilitation programs for individuals with post-COVID conditions is highlighted in an evidence-based position paper, which outlines recommendations for pulmonary, aerobic, and resistance training sessions tailored to patients’ specific needs [61]. One of the recommendations includes a rehabilitation treatment plan consisting of pulmonary, aerobic, and resistance training sessions three times per week for 6–8 weeks for individuals experiencing post-COVID conditions. Such personalized approaches are essential for optimizing outcomes and promoting long-term recovery. Rehabilitation programs for individuals with persistent post-COVID-19 symptoms should be tailored to their specific needs, given the diverse array of symptoms and impairments they may present. Glöckl et al. [62] emphasize the importance of categorizing patients into non-organ-specific phenotypes, which include those with mild COVID-19 but experiencing multi-organ sequelae, individuals with myalgic encephalomyelitis or chronic fatigue syndrome, those with severe-to-critical COVID-19 and post-intensive care syndrome, and those experiencing the unmasking and/or exacerbation of underlying comorbidities (black box). This approach of person-oriented segmentation seems to be necessary for guiding treatment planning more effectively. However, further research is needed to establish optimal care pathways. Group differences were observed in the improvement of physical performance between younger and older post-COVID patients, mild/moderate COVID-19 and severe/critical COVID-19, and patients with and without pre-existing cardiovascular disease, metabolic disease, psychological disease, neuro-sensory, musculoskeletal disease, repeated rehabilitation, and exercising in an outpatient group. Despite the small number of individuals with pre-existing psychological conditions in our study, there was a higher prevalence of these conditions compared to the general population [63]. Nevertheless, these findings underscore the importance of considering individual characteristics and comorbidities in tailoring rehabilitation interventions to meet the specific needs of patients with post-COVID conditions. Our results also showed significant differences in physical performance 12 months after rehabilitation depending on individual implementation of aftercare interventions, e.g., repeated rehabilitation or exercising in an outpatient group. Both of these treatments appear to be useful in supporting the recovery after post-COVID. This demonstrates the need to empower patients to enhance self-management and resources for health behaviours. Further research is needed to identify successful aftercare strategies that take into account the diversity as well as the individual course of post-COVID.

Additionally, telerehabilitation should also be considered as a valuable option. Telerehabilitation offers a flexible and accessible modality of care, particularly in the context of ongoing public health challenges and restrictions. Results from a randomized controlled trial support the efficacy of supervised home telerehabilitation program in improving physical performance, strength, and mechanical efficiency in patients with post-COVID sequelae [64].

Moreover, post-COVID is associated with poorer subjective financial well-being, sometimes with existential fears and an increased likelihood of making new benefit claims, highlighting the broader socio-economic impact of the pandemic [65]. Income and productivity loss exacerbate these challenges [66,67]. Given these complexities, it becomes important for healthcare systems and policymakers to adopt a holistic approach to post-recovery care. This includes not only addressing the physical and mental health aspects of post-COVID conditions but also recognizing and addressing the socio-economic factors that impact individuals’ well-being. Implementing initiatives to support financial stability, such as financial counselling and social welfare programs, can play a crucial role in promoting recovery in a long-term and enhancing the overall quality of life of those affected by post-COVID.

### Limitations

Despite the promising findings, our study has some limitations that warrant consideration. It is important to approach the results with caution, considering the observational nature of the study design and the absence of a control group taken into account that post-COVID patients did not reach normal value in physical performance by natural recovery [68]. Additionally, the relatively small sample size (and the specific population of health care workers) may compromise the generalisability of our findings to broader populations. Moreover, reliance on self-reported measures introduces the potential for measurement error and subjective bias.

Additionally, our investigation focused on a specific study population with work-related SARS-CoV-2 infection attending an inpatient rehabilitation at only one clinic, potentially introducing bias in participant recruitment, and affecting the representativeness of the study population. It is also noteworthy that overall, only 1.7% of the patients had no comorbidity prior to COVID-19. This high prevalence of pre-existing conditions may limit the applicability of our findings to healthier populations and underscores the need for further research in more diverse cohorts.

Furthermore, the variability in physical performance tests used to evaluate post-COVID exercise capacity poses challenges for cross-study comparisons and synthesis of findings [69]. Standardization of assessment protocols and outcome measures is essential for advancing our understanding of post-COVID rehabilitation outcomes and facilitating evidence-based practice. The dynamic nature of the pandemic, exemplified by the emergence of new COVID-19 variants like Omicron, underscores the need for ongoing surveillance and adaptation of healthcare strategies. The development of new variants after our study began has limited our ability to fully understand their differential effects on physical health. This oversight highlights the importance of considering evolving epidemiological factors in future research endeavours.

Moreover, it is important to note that all recruited patients started their rehabilitation until summer of 2022, when the alpha and delta variants were predominant. The emergence of the omicron variant has since led to a shift in the epidemiology of post-COVID conditions. Data from the British Symptom Study App suggest a reduction in the incidence of post-COVID by approximately 25–50% compared to the delta variant [70]. This suggests that later variants may have less impact on post-recovery outcomes.

## 5. Conclusions

In conclusion, our study highlights the persistent challenges faced by individuals recovering from COVID-19, including enduring symptoms like exertional intolerance and fatigue 12 months after rehabilitation. Despite these obstacles, significant improvements in physical performance were noted, indicating the potential benefits of a multidisciplinary rehabilitation program. However, the study’s limitations and the evolving nature of the pandemic underscore the need for further research and standardized approaches to post-COVID rehabilitation. Personalized rehabilitation plans will be essential for addressing individual needs and optimizing outcomes. Overall, our findings emphasize the importance of comprehensive care strategies to support individuals in their recovery journey, particularly over the 6- and 12-month post-rehabilitation periods.

## Figures and Tables

**Table 1:** Sociodemographic data at T1.

	N = 121 ^1^	Mean	SD ^2^	NA ^3^
Sex				-
Male	29 (24%)			
Female	92 (76%)			
Age [years]		50.62	10.87	
BMI [kg/m^2^]				-
Normal	17 (14%)			
Overweight	39 (32%)			
Obesity class I	35 (29%)			
Obesity class II	20 (17%)			
Obesity class III	10 (8%)			
Smoking status				-
Currently (every day)	5 (4%)			
Currently (occasional)	4 (3%)			
Former	46 (38%)			
Never	66 (55%)			
COVID-19 severity				-
Mild/Moderate	85 (70%)			
Severe	30 (25%)			
Critical	6 (5%)			
Hospitalisation due to COVID-19	32 (26%)			-
Hospitalisation [days]		13.86	19.32	
ICU care	9 (7%)			
Pneumonia due to COVID-19	36 (30%)			1
Interval COVID-19—Rehabilitation [days]		409.52	143.97	-
Rehabilitation duration [days]		28.99	5.22	-
PCFS score before Rehabilitation				-
No limitations	11 (9%)			
Negligible limitations	2 (2%)			
Slight limitations	43(36%)			
Moderate limitations	63 (52%)			
Severe limitations	2 (2%)			
Occupation				-
Healthcare worker	85 (70%)			
Non-healthcare worker	36 (30%)			
Socio-economic status				1
Medium	41 (34%)			
High	79 (66%)			
Pre-existing conditions				-
Metabolic disease	79 (65%)			
Cardiovascular disease	58 (48%)			
Respiratory disease	52 (43%)			
Psychological disease	23 (19%)			
Neuro-sensory disease	40 (33%)			
Musculoskeletal disease	80 (66%)			
Aftercare interventions				
Further medical treatment	96 (79%)			9
Repeated rehabilitation	28 (23%)			10
Exercising in an outpatient group	18 (15%)			16
Ambulatory physiotherapy	95 (79%)			11

^1^ n (%); ^2^ SD = standard deviation; ^3^ NA = not available/no answer.

**Table 2:** Differences in physical health between T1 and T3.

		T1			T3					
	n	Min	Max	Median (IQR)	Min	Max	Median (IQR)	Z	*p*	r
6MWD [m]	108	202.00	701.00	519.50 (448.50–575.25)	310.00	766.00	588.00 (519.00–644.00)	−7.841	<0.001 **	−0.755
Gait velocity [m/s]	108	0.84	2.10	1.48 (1.33–1.66)	1.11	2.53	1.69 (1.51–1.88)	−7.113	<0.001 **	−0.684
Watt_max_ [W]	50	32.00	288.00	96.50 (76.25–113.00)	26.00	324.00	108.00 (88.75–136.25)	−3.350	<0.001 **	−0.474
VO2_max_ [L/Min]	50	0.66	3.29	1.44 (1.21–1.56)	0.69	3.83	1.52 (1.34–1.81)	−3.316	<0.001 **	−0.469
1MSTS [n/Min]	107	9.00	43.00	20.00 (16.00–24.00)	9.00	45.00	21.00 (17.00–26.00)	−2.981	0.003 *	−0.288
Quadricep strength [kg]	108	33.02	252.09	97.78 (74.08–133.51)	28.83	268.34	109.09 (89.41–141.00)	−5.766	<0.001 **	−0.555
Handgrip strength [kg]	108	11.60	70.13	28.20 (21.32–35.06)	7.67	70.60	29.17 (22.87–35.17)	−0.884	0.377	−0.085
Balance function [0–36 points]	108	3.00	35.00	25.00 (21.25–27.00)	11.00	35.00	26.50 (22.00–30.00)	−3.072	0.002 *	−0.297
Subjective physical ability [0–10 points]	117	1.33	8.00	4.67 (3.44–6.00)	1.33	9.33	5.11 (3.78–6.44)	−3.514	<0.001 **	−0.325
PCFS [grade 0–4]	117	0.00	4.00	3.00 (2.00–3.00)	0.00	4.00	3.00 (2.00–3.00)	−0.266	0.790	−0.219
Subjective post-COVID symptoms [0–10 points]	117	1.00	10.00	7.00 (4.00–8.00)	0.00	10.00	7.00 (4.00–8.00)	−0.873	0.383	−0.081

IQR = interquartile range. * *p* < 0.05. ** *p* < 0.001.

**Table 3:** Differences in physical health between T1 and T4.

		T1			T4					
	n	Min	Max	Median (IQR)	Min	Max	Median (IQR)	Z	*p*	r
6MWD [m]	95	202.00	701.00	521.00 (455.00–581.00)	330.00	852.00	571.00 (502.00–628.00)	−6.635	<0.001 **	−0.681
Gait velocity [m/s]	95	0.99	2.10	1.47 (1.35–1.68)	0.92	2.51	1.65 (1.47–1.83)	−5.668	<0.001 **	−0.582
Watt_max_ [W]	51	37.00	177.00	97.00 (77.00–113.00)	30.00	190.00	108.00 (84.00–139.00)	−3.005	0.003 *	−0.421
VO2_max_ [L/Min]	51	0.96	2.27	1.41 (1.21–1.56)	0.71	2.43	1.50 (1.28–1.81)	−3.117	0.002 *	−0.437
1MSTS [n/Min]	96	8.00	43.00	20.00 (16.00–23.75)	5.00	42.00	21.00 (17.00–26.00)	−3.840	<0.001 **	−0.392
Quadricep strength [kg]	96	33.02	252.09	98.53 (74.08–134.20)	26.46	273.45	115.78 (87.47–158.79)	−5.525	<0.001 **	−0.564
Handgrip strength [kg]	98	9.67	70.13	27.47 (20.51–35.50)	7.97	67.57	28.92 (21.16–35.68)	−0.085	0.932	−0.009
Balance function [0–36 points]	96	3.00	35.00	25.00 (22.00–27.00)	6.00	36.00	26.00 (23.00–29.00)	−2.552	0.011 *	−0.261
Subjective physical ability [0–10 points]	112	1.33	8.00	4.78 (3.47–6.11)	0.89	9.44	5.33 (4.03–6.56)	−3.496	<0.001 **	−0.330
PCFS [grade 0–4]	112	0.00	4.00	3.00 (2.00–3.00)	0.00	4.00	3.00 (2.00–3.00)	−1.997	0.046 *	−0.189
Subjective post-COVID symptoms [0–10 points]	112	1.00	10.00	7.00 (4.00–8.00)	0.00	10.00	6.00 (3.00–8.00)	−1.675	0.094	−0.158

IQR = interquartile range. * *p* < 0.05. ** *p* < 0.001.

## Data Availability

The data are available from the corresponding author upon request.

## Supplementary Materials

The following supporting information can be downloaded at: https://www.mdpi.com/article/10.3390/jcm13133988/s1, Table S1: Description of the multidisciplinary post-COVID rehabilitation program at the BG Hospital Bad Reichenhall; Table S2: Differences in physical health at T1, T3, T4 (Friedman test); Table S3: Groupwise comparison of physical health of male and female post-COVID patients between timepoints T1 and T3; Table S4: Groupwise comparison of physical health of male and female post-COVID patients between timepoints T1 and T4; Table S5: Groupwise comparison of physical health of younger and older post-COVID patients between timepoints T1 and T3; Table S6: Groupwise comparison of physical health of younger and older post-COVID patients between timepoints T1 and T4; Table S7: Groupwise comparison of physical health of patients with mild-moderate COVID-19 and severe-critical between timepoints T1 and T3; Table S8: Groupwise comparison of physical health of patients with mild-moderate COVID-19 and severe-critical between timepoints T1 and T4; Table S9: Groupwise comparison of physical health of patients with pre-existing cardiovascular disease and without a pre-existing cardiovascular disease between timepoints T1 and T3; Table S10: Groupwise comparison of physical health of patients with pre-existing cardiovascular disease and without a pre-existing cardiovascular disease between timepoints T1 and T4; Table S11: Groupwise comparison of physical health of patients with pre-existing respiratory disease and without a pre-existing respiratory disease between timepoints T1 and T3; Table S12: Groupwise comparison of physical health of patients with pre-existing respiratory disease and without a pre-existing respiratory disease between timepoints T1 and T4; Table S13: Groupwise comparison of physical health of patients with pre-existing metabolic disease and without a pre-existing metabolic disease between timepoints T1 and T3; Table S14: Groupwise comparison of physical health of patients with pre-existing metabolic disease and without a pre-existing metabolic disease between timepoints T1 and T4; Table S15: Groupwise comparison of physical health of patients with pre-existing psychological disease and without a pre-existing psychological disease timepoints T1 and T3; Table S16: Groupwise comparison of physical health of patients with pre-existing psychological disease and without a pre-existing psychological disease timepoints T1 and T4; Table S17: Groupwise comparison of physical health of patients with pre-existing neuro-sensory disease and without a pre-existing neuro-sensory disease between timepoints T1 and T3; Table S18: Groupwise comparison of physical health of patients with pre-existing neuro-sensory disease and without a pre-existing neuro-sensory disease between timepoints T1 and T4; Table S19: Groupwise comparison of physical health of patients with pre-existing musculoskeletal disease and without a pre-existing musculoskeletal disease between timepoints T1 and T3; Table S20: Groupwise comparison of physical health of patients with pre-existing musculoskeletal disease and without a pre-existing musculoskeletal disease between timepoints T1 and T4; Table S21: Groupwise comparison of physical health of patients with repeated rehabilitation and without repeated rehabilitation between timepoints T1 and T4; Table S22: Groupwise comparison of physical health of patients with exercising in an outpatient group and those not exercising in an outpatient group between timepoints T1 and T4.
