# Supplementary material for: Physical Health in Patients with Post-COVID-19 6 and 12 Months after an Inpatient Rehabilitation: An Observational Study
Source: J Clin Med. 2024 Jul 8;13(13):3988. doi: 10.3390/jcm13133988 (PMC11242682; doi:10.3390/jcm13133988)
Supplement: Supplementary file 1 [file jcm-13-03988-s001.zip › jcm-3093382-supplementary.pdf]

Table S1. Description of the multidisciplinary post-COVID rehabilitation program at the BG Hospital Bad Reichenhall.

Initial illness specific diagnostic and pharmacological treatment	• Conducted by professionals<br>• Continuous adaptation in the course of rehabilitation
Psychological therapy	• After initial psychological interviews with the patients at the beginning of the rehabilitation, the patients participate in the following four education groups once a week, as recommended by a psychologist: "Anxiety", "Depression", "Sleep & Stress", "Cognitive Training"<br>• Weekly group meetings for mindfulness, art therapy, and post-COVID-19 group therapy, automatically scheduled for all patients<br>• Individual psychological and psychiatric sessions will be conducted if needed
Cognitive Training	• Cognitive Training is pre-discussed individually and is carried out by the patients twice a week for 10–15 min using the computer-based program "Fresh Minder 2, 3, and 4" (Version 2.7.7, Version 3.5.5, Version 4.0.8, Ditzungen, Germany)
Sports therapy/ Physiotherapy/ respiratory therapy	• After assessing initial physical condition (cardiovascular und motor fitness), patients receive an individual training program with sports therapy and physiotherapy.<br>The program consists of:<br>• endurance training (e.g., Nordic walking, ergometer training), strength training (e.g., medical training therapy, stair climbing exercise), training of coordination, mobility and fascia training, relaxation exercises (e.g., progressive muscles relaxation)<br>• respiratory physiotherapy (e.g., mobility and respiratory techniques, inspiratory muscle training, specific manual therapy)
Health promotion interventions	• E.g., smoke cessation, stress management, physical activity

Table S2: Differences in physical health at T1, T3, T4 (Friedman test).

	Median	IQR	X^2	p
		6MWD [m]		
T1	523.00	463.00 - 582.25		
T2	596.00	527.25 - 656.75	74.81	<0.001**
T3	578.00	507.75 - 637.25		
		Gait velocity [m/s]		
T1	1.48	1.35 - 1.68		
T2	1.72	1.52 - 1.90	56.60	<0.001**
T3	1.67	1.49 - 1.67		

	Median	IQR	X^2	p
		$Watt_{max}$ [W]		
T1	101.00	77.00 - 113.00		
T2	108.00	95.00 - 137.00	14.05	<0.001**
T3	114.00	87.00 - 141.00		
		$VO2_{max}$ [l/Min]		
T1	1.45	1.22 - 1.56		
T2	1.53	1.38 - 1.81	12.61	0.002
T3	1.56	1.35 - 1.84		
		1MSTS [n/Min]		
T1	20.00	16.00 - 24.00		
T2	21.00	18.00 - 26.00	15.08	<0.001**
T3	21.00	18.00 - 26.00		
		Quadriceps strength [kg]		
T1	99.79	75.25 135.32		
T2	109.09	92.19 144.07	41.89	<0.001**
T3	116.30	88.18 159.93		
		Handgrip strength [kg]		
T1	27.90	21.03 - 35.50		
T2	29.33	21.62 - 35.62	0.67	0.717
T3	29.03	21.61 - 36.28		
		Balance function [0-36 points]		
T1	25.00	22.00 - 27.00		
T2	27.00	22.00 - 30.00	17.37	<0.001**
T3	26.00	23.00 - 30.00		
		Subjective physical ability [0-10 points]		
T1	4.78	3.56 - 6.19		
T2	5.33	4.00 - 6.56	14.60	<0.001**
T3	5.39	4.22 - 6.56		
		PCFS [grade 0-4]		
T1	3,00	2,00 - 3,00		
T2	3,00	2,00 - 3,00	4.194	0.124
T3	2,00	2,00 - 3,00		
		Subjective post-COVID symptoms [0-10 points]		
T1	3.00	2.00 - 6.00		
T2	3.00	2.00 - 6.00	0.99	0.610
T3	4.00	2.00 - 7.00		

IQR = interquartile range. *p<0.05. **p<0.001.

Table S3: Groupwise comparison of physical health of male and female post-COVID patients between timepoints T1 and T3.

	Male				Female				Between-group difference		
	N	T1 Median (IQR)	T3 Median (IQR)	Δ	N	T1 Median (IQR)	T3 Median (IQR)	Δ	z	p	r
6MWD [m]	28	532.00 (464.25 – 583.75)	608.50 (521.75 – 675.25)	78.50 (29.50 – 122.00)	80	517.00 (434.00 – 559.75)	579.00 (515.25 – 637.00)	70.00 (21.25 – 111.75)	-0.673	0.501	-0.065
Gait velocity [m/s]	28	1.54 (1.37 – 1.71)	1.79 (1.47 – 2.00)	0.24 (0.04 – 0.43)	80	1.47 (1.31 – 1.65)	1.67 (1.51 – 1.85)	0.20 (0.04 – 0.35)	-0.561	0.575	-0.054
$Watt_{max}$ [W]	16	114.00 (88.25 – 148.00)	133.50 (95.50 – 181.50)	22.00 (-7.00 – 34.25)	34	92.50 (72.75 – 108.00)	105.50 (86.75 – 122.25)	11.00 (-4.00 – 28.00)	-0.718	0.473	-0.102
$VO2_{max}$ [l/Min]	16	1.63 (1.40 – 2.13)	1.85 (1.33 – 2.34)	0.18 (-0.05 – 0.37)	34	1.35 (1.17 – 1.53)	1.46 (1.34 – 1.61)	0.13 (-0.03 – 0.27)	-0.218	0.827	-0.031
1MSTST [n/Min]	28	18.50 (15.00 – 22.75)	18.50 (16.00 – 24.75)	1.00 (-2.00 – 4.00)	79	21.00 (17.00 – 24.00)	22.00 (18.00 – 26.00)	1.00 (-1.00 – 5.00)	0.673	0.501	0.065
Quadriceps strength [kg]	28	136.29 (120.09 – 147.39)	138.24 (123.88 – 138.99)	7.56 (0.87 – 33.28)	80	87.47 (68.68 – 114.17)	103.05 (86.53 – 127.10)	11.91 (-1.05 – 25.93)	0.147	0.883	0.014
Handgrip strength [kg]	28	38.35 (32.33 – 49.15)	41.23 (30.67 – 49.72)	0.12 (-7.27 – 5.45)	80	25.13 (20.30 – 31.54)	26.38 (22.21 – 30.78)	1.03 (-2.43 – 3.61)	0.291	0.771	0.028
Balance function [0-36 points]	28	25.00 (22.00 – 27.00)	26.50 (22.50 – 30.00)	2.00 (-2.50 – 5.00)	80	25.00 (20.25 – 27.00)	26.50 (22.00 – 29.00)	2.00 (-1.00 – 5.00)	-0.011	0.992	-0.001
Subjective physical ability [0-10 points]	28	5.56 (3.61 – 7.00)	5.78 (4.72 – 7.44)	0.67 (-0.64 – 1.69)	89	4.67 (3.17 – 5.44)	5.00 (3.78 – 6.17)	0.33 (-0.33 – 1.56)	-0.134	0.893	-0.012
PCFS [grade 0-4]	28	2.00 (2.00 - 3.00)	2.50 (0.50 - 3.00)	0.00 (0.00 – 0.00)	89	3.00 (2.00 – 3.00)	3.00 (2.00 – 3.00)	0.00 (0.00 – 0.00)	-0.290	0.977	-0.027
Subjective post-COVID symptoms [0-10 points]	28	3.00 (2.00 – 6.00)	3.00 (2.00 – 6.00)	0.00 (-1.75 – 2.75)	89	2.00 (1.50 – 6.00)	3.00 (2.00 – 6.00)	0.00 (-2.00 – 2.00)	-0.594	0.552	-0.055

IQR = interquartile range.

Table S4: Groupwise comparison of physical health of male and female post-COVID patients between timepoints T1 and T4.

	Male				Female				Between-group difference		
	N	T1 Median (IQR)	T4 Median (IQR)	Δ	N	T1 Median (IQR)	T4 Median (IQR)	Δ	z	p	r
6MWD [m]	26	519.50 (457.50 – 583.25)	613.00 (490.25 – 646.75)	69.00 (28.00 – 107.50)	69	522.00 (451.50 – 565.50)	562.00 (503.00 – 606.50)	44.00 (5.00 – 79.50)	-1.565	0.118	-0.161
Gait velocity [m/s]	26	1.44 (1.35 – 1.69)	1.68 (1.46 – 1.86)	0.17 (0.05 – 0.33)	69	1.49 (1.34 – 1.67)	1.65 (1.48 – 1.83)	0.14 (0.02 – 0.31)	-0.309	0.757	-0.032
$Watt_{max}$ [W]	15	109.00 (88.00 – 142.00)	139.00 (96.00 – 153.00)	21.00 (8.00 – 29.00)	36	94.00 (73.25 – 111.25)	104.00 (81.25 – 127.00)	11.00 (-1.25 – 28.00)	-0.910	0.449	-0.127
$VO2_{max}$ [l/Min]	15	1.47 (1.38 – 1.97)	1.84 (1.41 – 2.18)	0.23 (0.04 – 0.39)	36	1.32 (1.18 – 1.52)	1.47 (1.18 – 1.72)	0.09 (-0.02 – 0.25)	-1.137	0.256	-0.159
1MSTST [n/Min]	26	18.50 (14.75 – 22.25)	20.00 (17.75 – 26.00)	2.00 (-0.25 – 5.00)	70	20.00 (16.00 – 24.00)	21.00 (18.00 – 26.25)	2.00 (-1.25 – 5.00)	-0.351	0.725	-0.036
Quadriceps strength [kg]	26	134.99 (115.54 – 145.81)	157.87 (111.30 – 180.65)	15.35 (-13.67 – 42.46)	70	87.47 (67.38 – 115.92)	105.36 (79.52 – 132.88)	19.02 (2.10 – 37.06)	0.305	0.760	0.031
Handgrip strength [kg]	26	38.35 (33.05 – 49.21)	40.08 (31.18 – 46.17)	-1.85 (-8.60 – 2.78)	72	24.53 (20.08 – 30.83)	25.80 (19.99 – 32.18)	0.55 (-3.78 – 3.92)	1.412	0.158	0.143
Balance function [0-36 points]	26	24.50 (22.00 – 27.25)	26.00 (22.25 – 30.00)	2.00 (-1.25 – 4.00)	70	25.00 (20.75 – 27.00)	26.00 (23.00 – 29.00)	1.00 (-1.00 – 3.00)	-0.356	0.722	-0.036
Subjective physical ability [0-10 points]	28	5.72 (4.17 – 7.00)	6.17 (4.69 – 7.33)	0.56 (-0.36 – 1.17)	84	4.67 (3.28 – 5.44)	4.94 (3.69 – 6.44)	0.61 (-0.56 – 1.56)	0.239	0.811	0.023

Table S4: Groupwise comparison of physical health of male and female post-COVID patients between timepoints T1 and T4.

	Male				Female				Between-group difference		
	N	T1 Median (IQR)	T4 Median (IQR)	Δ	N	T1 Median (IQR)	T4 Median (IQR)	Δ	z	p	r
PCFS [grade 0-4]	28	2.00 (2.00 - 3.00)	2.00 (0.00 - 3.00)	0.00 (0.00 – 0.00)	84	3.00 (2.00 – 3.00)	3.00 (2.00 – 3.00)	0.00 (-0.75 – 0.00)	-0.517	0.605	-0.049
Subjective post-COVID symptoms [0-10 points]	28	3.00 (2.00 – 6.00)	5.00 (2.25 – 6.75)	0.50 (-1.00 – 3.75)	84	2.50 (2.00 – 6.00)	3.00 (2.00 – 7.00)	0.00 (-1.00 – 2.00)	-0.962	0.336	-0.091

IQR = interquartile range.

Table S5: Groupwise comparison of physical health of younger and older post-COVID patients between timepoints T1 and T3.

	younger than 51 years				at least 51 years				Between-group difference		
	N	T1 Median (IQR)	T3 Median (IQR)	Δ	N	T1 Median (IQR)	T3 Median (IQR)	Δ	z	p	r
6MWD [m]	40	541.50 (461.00 – 579.75)	608.00 (552.25 – 666.50)	74.00 (32.00 – 128.00)	68	517.00 (436.50 – 570.00)	576.00 (502.25 – 643.00)	71.50 (14.50 – 115.25)	-1.174	0.240	-0.113
Gait velocity [m/s]	40	1.54 (1.37 – 1.71)	1.78 (1.62 – 1.94)	0.22 (0.04 – 0.41)	68	1.45 (1.24 – 1.66)	1.65 (1.41 – 1.87)	0.17 (0.03 – 0.35)	-0.900	0.368	-0.087
$Watt_{max}$ [W]	15	108.00 (77.00 – 150.00)	108.00 (95.00 – 137.00)	13.00 (1.00 – 28.00)	35	95.00 (73.00 – 109.00)	108.00 (83.00 – 130.00)	16.00 (-4.00 – 31.00)	0.611	0.508	0.086
$VO2_{max}$ [l/Min]	15	1.43 (1.17 – 1.96)	1.52 (1.38 – 1.74)	0.16 (0.03 – 0.22)	35	1.45 (1.21 – 1.55)	1.51 (1.32 – 1.83)	0.13 (-0.03 – 0.36)	0.275	0.783	0.039
1MSTST [n/Min]	40	21.00 (19.25 – 25.75)	24.50 (19.50 – 30.75)	2.00 (-1.00 – 5.75)	67	19.00 (15.00 – 23.00)	19.00 (16.00 – 23.00)	0.00 (-2.00 – 4.00)	-0.993	0.321	-0.096
Quadriceps strength [kg]	40	107.98 (76.40 – 135.70)	125.06 (101.60 – 144.58)	15.33 (3.19 – 37.05)	68	94.16 (72.13 – 132.08)	103.57 (81.19 – 138.49)	7.71 (-1.60 – 18.51)	-2.087	0.037	-0.201
Handgrip strength [kg]	40	32.97 (24.95 – 36.08)	29.95 (23.45 – 36.72)	0.27 (-2.69 – 2.37)	68	25.13 (20.10 – 32.38)	28.57 (22.13 – 32.87)	1.03 (-3.53 – 4.58)	0.719	0.472	0.069
Balance function [0-36 points]	40	27.00 (23.25 – 29.75)	29.00 (25.00 – 32.75)	2.00 (-1.00 – 5.00)	68	24.50 (20.00 – 26.00)	26.00 (21.00 – 28.00)	1.00 (-1.75 – 5.00)	-0.520	0.603	-0.050
Subjective physical ability [0-10 points]	44	4.78 (3.69 – 5.97)	5.56 (4.33 – 6.89)	0.72 (-0.33 – 1.64)	73	4.67 (3.28 – 6.22)	4.89 (3.56 – 6.00)	0.22 (-0.33 – 1.50)	-0.898	0.369	-0.083
PCFS [grade 0-4]	44	3.00 (2.00 – 3.00)	3.00 (2.00 – 3.00)	0.00 (0.00 – 0.00)	73	3.00 (2.00 – 3.00)	3.00 (2.00 – 3.00)	0.00 (0.00 – 0.00)	0.455	0.649	0.042
Subjective post-COVID symptoms [0-10 points]	44	2.00 (1.00 - 5.00)	3.50 (1.25 – 6.00)	1.00 (-1.00 – 2.75)	73	3.00 (2.00 – 6.50)	3.00 (2.00 – 6.00)	0.00 (-2.00 – 2.00)	-1.570	0.116	-0.145

IQR = interquartile range.

Table S6: Groupwise comparison of physical health of younger and older post-COVID patients between timepoints T1 and T4.

	younger than 51 years				at least 51 years				Between-group difference		
	N	T1 Median (IQR)	T4 Median (IQR)	Δ	N	T1 Median (IQR)	T4 Median (IQR)	Δ	z	p	r
6MWD [m]	33	546.00 (466.50 – 583.00)	596.00 (544.50 – 640.00)	46.00 (21.00 – 84.00)	62	519.50 (443.50 – 566.25)	550.00 (483.50 – 622.00)	46.50 (1.50 – 85.75)	-0.340	0.734	-0.035
Gait velocity [m/s]	33	1.54 (1.38 – 1.76)	1.72 (1.60 – 1.94)	0.24 (-0.02 – 0.38)	62	1.45 (1.24 – 1.65)	1.62 (1.44 – 1.83)	0.13 (0.04 – 0.29)	-0.918	0.358	-0.094
$Watt_{max}$ [W]	16	106.00 (74.75 – 142.00)	116.00 (85.50 – 139.50)	10.00 (-0.25 – 27.00)	35	96.00 (77.00 – 112.00)	103.00 (84.00 – 139.00)	14.00 (1.00 – 29.00)	0.467	0.640	0.065
$VO2_{max}$ [l/Min]	16	1.41 (1.16 – 1.86)	1.57 (1.27 – 1.74)	0.15 (-0.01 – 0.26)	35	1.41 (1.21 – 1.53)	1.49 (1.20 – 1.84)	0.09 (-0.01 – 0.38)	0.203	0.839	0.028
1MSTST [n/Min]	33	21.00 (19.00 – 26.50)	24.00 (20.00 – 34.00)	2.00 (0.00 – 5.00)	63	18.00 (15.00 – 23.00)	20.00 (16.00 – 24.00)	2.00 (-2.00 – 5.00)	-0.789	0.430	-0.081
Quadriceps strength [kg]	33	109.31 (84.37 – 139.30)	129.23 (102.92 – 179.65)	28.66 (6.66 – 44.54)	63	91.93 (69.60 – 131.90)	104.07 (80.25 – 156.93)	16.42 (-1.21 – 32.42)	-1.894	0.058	-0.193
Handgrip strength [kg]	33	33.53 (25.27 – 37.02)	32.67 (22.50 – 37.53)	-0.07 (-4.02 – 3.47)	65	24.43 (20.00 – 32.83)	27.97 (20.23 – 34.78)	0.20 (-4.92 – 4.08)	0.143	0.886	0.014
Balance function [0-36 points]	33	27.00 (24.00 – 29.50)	29.00 (26.00 – 31.00)	2.00 (-1.00 – 3.00)	63	24.00 (20.00 – 26.00)	25.00 (22.00 – 27.00)	1.00 (-2.00 – 4.00)	-0.255	0.798	-0.026
Subjective physical ability [0-10 points]	42	4.83 (4.19 – 6.00)	5.44 (4.42 – 6.72)	0.56 (-0.56 – 1.47)	70	4.72 (3.31 – 6.22)	5.11 (3.56 – 6.47)	0.56 (-0.36 – 1.56)	-0.114	0.909	-0.011
PCFS [grade 0-4]	42	3.00 (2.00 – 3.00)	3.00 (2.00 – 3.00)	0.00 (-0.25 – 0.00)	70	2.50 (2.00 – 3.00)	2.50 (1.75 – 3.00)	0.00 (-0.25 – 0.00)	0.453	0.651	0.043
Subjective post-COVID symptoms [0-10 points]	42	2.00 (1.00 – 5.00)	3.00 (2.00 – 6.00)	0.50 (-1.00 – 2.00)	70	3.00 (2.00 – 6.00)	4.50 (2.00 – 7.00)	0.00 (-1.00 – 3.00)	-0.464	0.643	-0.044

IQR = interquartile range.

Table S7: Groupwise comparison of physical health of patients with mild-moderate COVID-19 and severe-critical between timepoints T1 and T3.

	mild/moderate				severe/critical				Between-group difference		
	N	T1 Median (IQR)	T3 Median (IQR)	Δ	N	T1 Median (IQR)	T3 Median (IQR)	Δ	z	p	r
6MWD [m]	74	539.50 (466.75 – 584.25)	605.00 (549.75 – 661.50)	71.50 (22.75 – 116.50)	34	494.50 (432.50 – 536.25)	545.00 (503.50 – 606.25)	74.00 (19.75 – 115.00)	-0.195	0.845	-0.019
Gait velocity [m/s]	74	1.51 (1.35 – 1.70)	1.74 (1.54 – 1.89)	0.20 (0.08 – 0.38)	34	1.43 (1.24 – 1.60)	1.62 (1.40 – 1.82)	0.19 (0.02 – 0.42)	-0.284	0.776	-0.027
$Watt_{max}$ [W]	31	104.00 (78.00 – 128.00)	118.00 (95.00 – 148.00)	20.00 (6.00 – 28.00)	19	88.00 (72.00 – 109.00)	97.00 (80.00 – 111.00)	-1.00 (-5.00 – 31.00)	-0.940	0.347	-0.133
$VO2_{max}$ [l/Min]	31	1.45 (1.22 – 1.79)	1.53 (1.41 – 1.89)	0.20 (0.02 – 0.30)	19	1.36 (1.15 – 1.48)	1.36 (1.27 – 1.60)	0.01 (-0.05 – 0.37)	-0.870	0.385	-0.123
1MSTST [n/Min]	73	21.00 (17.00 – 24.50)	23.00 (18.00 – 27.50)	1.00 (-2.00 – 5.00)	34	17.50 (14.00 – 22.00)	18.50 (16.00 – 22.00)	0.50 (-1.00 – 4.00)	-0.564	0.572	-0.055
Quadriceps strength [kg]	74	94.43 (73.95 – 132.37)	115.89 (94.86 – 140.77)	13.85 (2.30 – 30.11)	34	103.59 (73.05 – 134.62)	102.57 (79.25 – 143.29)	5.94 (-2.43 – 19.08)	-1.614	0.107	-0.155
Handgrip strength [kg]	74	28.57 (21.48 – 35.35)	29.37 (23.38 – 34.77)	0.38 (-2.61 – 3.73)	34	26.90 (20.46 – 34.41)	26.81 (21.93 – 35.89)	1.55 (-4.07 – 4.65)	0.466	0.641	0.045
Balance function [0-36 points]	74	25.00 (21.75 – 28.00)	26.50 (22.00 – 30.00)	2.00 (-2.00 – 5.00)	34	25.00 (20.75 – 27.00)	26.50 (19.50 – 30.00)	2.00 (-0.25 – 5.00)	0.404	0.686	0.039
Subjective physical ability [0-10 points]	82	4.78 (3.53 – 6.00)	5.33 (4.33 – 6.47)	0.39 (-0.33 – 1.56)	35	4.56 (3.11 – 6.11)	4.33 (3.33 – 6.33)	0.56 (-0.33 – 1.56)	-0.205	0.837	-0.019

Table S7: Groupwise comparison of physical health of patients with mild-moderate COVID-19 and severe-critical between timepoints T1 and T3.

	mild/moderate				severe/critical				Between-group difference		
	N	T1 Median (IQR)	T3 Median (IQR)	Δ	N	T1 Median (IQR)	T3 Median (IQR)	Δ	z	p	r
PCFS [grade 0-4]	82	2.50 (2.00 - 3.00)	2.50 (2.00 - 3.00)	0.00 (0.00 – 0.00)	35	3.00 (2.00 – 3.00)	3.00 (2.00 – 3.00)	0.00 (0.00 – 0.00)	1.361	0.174	0.126
Subjective post-COVID symptoms [0-10 points]	82	2.00 (1.00 – 5.00)	4.00 (2.00 – 6.00)	1.00 (-1.00 – 3.00)	35	5.00 (2.00 – 7.00)	3.00 (1.00 – 6.00)	-1.00 (-4.00 – 1.00)	-2.628	0.009	-0.243

IQR = interquartile range.

Table S8: Groupwise comparison of physical health of patients with mild-moderate COVID-19 and severe-critical between timepoints T1 and T4.

	mild/moderate				severe/critical				Between-group difference		
	N	T1 Median (IQR)	T4 Median (IQR)	Δ	N	T1 Median (IQR)	T4 Median (IQR)	Δ	z	p	r
6MWD [m]	66	531.00 (466.75 – 589.00)	584.00 (503.50 – 637.25)	45.50 (5.75 – 82.50)	29	499.00 (448.50 – 543.00)	540.00 (496.50 – 623.00)	46.00 (21.50 – 87.00)	0.473	0.636	0.049
Gait velocity [m/s]	66	1.50 (1.36 – 1.70)	1.68 (1.48 – 1.83)	0.14 (0.01 – 0.30)	29	1.43 (1.25 – 1.58)	1.61 (1.43 – 1.82)	0.17 (0.05 – 0.37)	0.974	0.330	0.100
$Watt_{max}$ [W]	34	105.00 (77.75 – 128.25)	122.00 (84.00 – 148.75)	14.50 (0.25 – 30.00)	17	88.00 (72.50 – 105.00)	99.00 (83.00 – 111.00)	13.00 (-4.00 – 28.50)	-0.370	0.712	-0.052
$VO2_{max}$ [l/Min]	34	1.45 (1.21 – 1.79)	1.60 (1.31 – 1.96)	0.10 (-0.01 – 0.38)	17	1.30 (1.17 – 1.48)	1.47 (1.18 – 1.66)	0.13 (-0.05 – 0.28)	-0.110	0.912	-0.015
1MSTST [n/Min]	66	20.50 (17.00 – 24.00)	21.50 (17.75 – 26.25)	2.00 (-2.00 – 5.00)	30	17.00 (14.00 – 22.25)	19.50 (17.00 – 24.25)	3.00 (0.00 – 5.25)	0.943	0.346	0.096
Quadriceps strength [kg]	66	92.68 (71.03 – 134.55)	113.98 (82.51 – 157.63)	17.89 (2.10 – 37.39)	30	105.98 (74.73 – 134.62)	116.47 (88.57 – 172.84)	22.64(-0.90 – 38.83)	0.292	0.770	0.030
Handgrip strength [kg]	68	27.27 (20.66 – 35.50)	29.25 (20.63 – 35.39)	0.47 (-3.78 – 4.14)	30	28.17 (20.16 – 35.88)	26.42 (21.77 – 37.11)	-0.80 (-6.45 – 3.13)	-0.964	0.335	-0.097
Balance function [0-36 points]	66	25.00 (22.00 – 28.00)	26.00 (23.00 – 30.00)	1.00 (-1.00 – 3.00)	30	24.50 (20.00 – 27.00)	25.00 (20.25 – 28.00)	0.50 (-2.00 – 4.25)	-0.420	0.674	-0.043
Subjective physical ability [0-10 points]	81	4.78 (3.83 – 6.06)	5.56 (4.22 – 6.56)	0.56 (-0.50 – 1.56)	31	4.56 (3.00 – 6.22)	4.78 (3.22 – 6.44)	0.78 (-0.78 – 1.44)	0.172	0.863	0.016
PCFS [grade 0-4]	81	2.00 (2.00 – 3.00)	2.00 (2.00 – 3.00)	0.00 (-1.00 – 0.00)	31	3.00 (2.00 – 3.00)	3.00 (2.00 – 3.00)	0.00 (0.00 – 0.00)	0.128	0.898	0.012
Subjective post-COVID symptoms [0-10 points]	81	3.00 (2.00 – 5.00)	3.00 (2.00 – 6.00)	0.00 (-1.00 – 2.00)	31	5.00 (2.00 – 7.00)	5.00 (2.00 – 9.00)	0.00 (-1.00 – 2.00)	0.364	0.716	0.034

IQR = interquartile range.

Table S9: Groupwise comparison of physical health of patients with pre-existing cardiovascular disease and without a pre-existing cardiovascular disease between timepoints T1 and T3.

	No				Yes				Between-group difference		
	N	T1 Median (IQR)	T3 Median (IQR)	Δ	N	T1 Median (IQR)	T3 Median (IQR)	Δ	z	p	r
6MWD [m]	58	540.00 (463.00 – 581.25)	590.50 (540.75 – 657.25)	70.50 (25.00 – 118.50)	50	496.50 (432.75 – 548.25)	580.00 (511.00 – 641.00)	76.50 (17.50 – 116.00)	-0.166	0.868	-0.016
Gait velocity [m/s]	58	1.54 (1.36 – 1.70)	1.69 (1.55 – 1.89)	0.20 (0.05 – 0.34)	50	1.44 (1.26 – 1.65)	1.68 (1.45 – 1.87)	0.20 (0.01 – 0.42)	0.046	0.963	0.004
$Watt_{max}$ [W]	29	101.00 (75.50 – 110.50)	105.00 (94.00 – 127.00)	15.00 (-4.50 – 27.00)	21	95.00 (74.50 – 116.00)	108.00 (81.50 – 155.50)	16.00 (-4.00 – 35.50)	0.993	0.321	0.140
$VO2_{max}$ [l/Min]	29	1.41 (1.20 – 1.56)	1.52 (1.35 – 1.75)	0.16 (-0.05 – 0.28)	21	1.46 (1.21 – 1.68)	1.47 (1.32 – 1.95)	0.13 (-0.03 – 0.38)	0.570	0.569	0.081
1MSTST [n/Min]	57	20.00 (17.00 – 24.50)	22.00 (18.00 – 27.00)	0.00 (-1.50 – 4.50)	50	19.50 (15.00 – 24.00)	19.50 (16.75 – 25.00)	1.00 (-1.25 – 4.00)	0.282	0.778	0.027
Quadriceps strength [kg]	58	92.59 (68.29 – 132.67)	107.51 (90.53 – 133.02)	9.94 (-1.31 – 25.47)	50	98.83 (83.80 – 134.39)	119.06 (88.11 – 150.89)	12.69 (3.86 – 29.77)	0.856	0.392	0.083
Handgrip strength [kg]	58	28.60 (21.79 – 35.13)	26.63 (22.71 – 34.49)	0.98 (-2.66 – 3.72)	50	27.57 (20.63 – 35.05)	29.80 (23.23 – 37.62)	0.77 (-3.62 – 4.33)	0.330	0.742	0.032
Balance function [0-36 points]	58	26.00 (23.00 – 28.00)	27.00 (22.75 – 30.00)	2.00 (-2.25 – 5.00)	50	23.00 (19.75 – 27.00)	26.00 (20.25 – 29.25)	2.00 (-1.00 – 5.25)	0.395	0.693	0.038
Subjective physical ability [0-10 points]	62	4.72 (4.00 – 5.75)	5.56 (4.33 – 6.61)	0.78 (-0.25 – 1.56)	55	4.67 (2.67 – 6.22)	4.89 (3.44 – 6.00)	0.22 (-0.78 – 1.56)	-0.759	0.448	-0.070
PCFS [grade 0-4]	62	3.00 (2.00 – 3.00)	3.00 (2.00 – 3.00)	0.00 (0.00 – 0.00)	55	3.00 (2.00 – 3.00)	3.00 (2.00 – 3.00)	0.00 (0.00 – 0.00)	0.069	0.945	0.006
Subjective post-COVID symptoms [0-10 points]	62	3.00 (2.00 – 6.25)	3.50 (2.00 – 6.00)	0.00 (-2.00 - 2.00)	55	2.00 (1.00 – 5.00)	3.00 (1.00 – 6.00)	0.00 (-1.00 – 2.00)	0.069	0.945	0.006

IQR = interquartile range.

Table S10: Groupwise comparison of physical health of patients with pre-existing cardiovascular disease and without a pre-existing cardiovascular disease between timepoints T1 and T4.

	No				Yes				Between-group difference		
	N	T1 Median (IQR)	T4 Median (IQR)	Δ	N	T1 Median (IQR)	T4 Median (IQR)	Δ	z	p	r
6MWD [m]	48	541.00 (471.25 – 582.75)	588.50 (522.00 – 635.50)	38.00 (3.00 – 98.00)	47	495.00 (432.00 – 544.00)	550.00 (482.00 – 621.00)	50.00 (31.00 – 81.00)	1.027	0.304	0.105
Gait velocity [m/s]	48	1.55 (1.39 – 1.72)	1.72 (1.53 – 1.82)	0.16 (-0.03 – 0.32)	47	1.44 (1.27 – 1.63)	1.61 (1.44 – 1.86)	0.17 (0.05 – 0.32)	0.331	0.740	0.034
$Watt_{max}$ [W]	30	98.50 (76.25 – 113.50)	111.00 (83.50 – 136.00)	12.50 (-6.25 – 25.75)	21	98.50 (76.25 – 113.50)	111.00 (83.50 – 136.00)	12.50 (-6.25 – 25.75)	0.919	0.358	0.129
$VO2_{max}$ [l/Min]	30	1.40 (1.20 – 1.56)	1.53 (1.19 – 1.81)	0.12 (-0.03 – 0.28)	21	1.45 (1.20 – 1.55)	1.47 (1.34 – 1.92)	0.09 (-0.00 – 0.39)	0.345	0.730	0.048
1MSTST [n/Min]	50	20.00 (15.00 – 23.25)	22.50 (18.00 – 27.25)	3.00 (-1.00 – 5.00)	46	19.50 (15.00 – 23.25)	20.00 (16.00 – 24.00)	1.50 (-1.00 – 4.00)	-1.140	0.254	-0.116
Quadriceps strength [kg]	49	98.46 (70.00 – 134.99)	116.89 (88.59 – 158.16)	28.66 (2.78 – 41.77)	47	98.60 (81.55 – 133.97)	109.17 (86.56 – 165.54)	13.05 (-1.21 – 27.33)	-2.129	0.033	-0.217
Handgrip strength [kg]	51	28.50 (20.83 – 35.50)	28.83 (21.03 – 35.40)	-0.07 (-3.87 – 4.63)	47	27.10 (20.20 – 35.50)	29.03 (21.77 – 36.13)	0.20 (-6.07 – 3.77)	-0.363	0.717	-0.037
Balance function [0-36 points]	50	25.50 (23.00 – 28.00)	26.00 (23.00 – 30.00)	1.00 (-1.00 – 4.00)	46	23.00 (20.00 – 27.00)	25.50 (21.50 – 28.25)	1.00 (-1.25 – 3.00)	-0.427	0.669	-0.044
Subjective physical ability [0-10 points]	59	4.78 (4.00 – 6.00)	5.33 (4.22 – 6.56)	0.56 (-0.33 – 1.44)	53	4.89 (3.11 – 6.28)	5.00 (3.67 – 6.56)	0.56 (-0.67 – 1.56)	-0.222	0.825	-0.021

Table S10: Groupwise comparison of physical health of patients with pre-existing cardiovascular disease and without a pre-existing cardiovascular disease between timepoints T1 and T4.

	No				Yes				Between-group difference		
	**N**	**T1 Median (IQR)**	**T4 Median (IQR)**	**Δ**	**N**	**T1 Median (IQR)**	**T4 Median (IQR)**	**Δ**	**z**	**p**	**r**
PCFS [grade 0-4]	59	3.00 (2.00 – 3.00)	3.00 (2.00 – 3.00)	0.00 (0.00 – 0.00)	53	2.00 (2.00 – 3.00)	2.00 (1.00 – 3.00)	0.00 (-1.00 – 0.00)	-0.396	0.692	-0.037
Subjective post-COVID symptoms [0-10 points]	59	3.00 (2.00 – 6.00)	4.00 (2.00 – 7.00)	1.00 (-1.00 – 2.00)	53	3.00 (2.00 – 6.00)	3.00 (1.50 – 6.00)	0.00 (-1.00 – 2.00)	-0.981	0.326	-0.093

IQR = interquartile range.

Table S11: Groupwise comparison of physical health of patients with pre-existing respiratory disease and without a pre-existing respiratory disease between timepoints T1 and T3.

	No				Yes				Between-group difference		
	**N**	**T1 Median (IQR)**	**T3 Median (IQR)**	**Δ**	**N**	**T1 Median (IQR)**	**T3 Median (IQR)**	**Δ**	**z**	**p**	**r**
6MWD [m]	61	521.00 (467.50 – 576.00)	587.00 (531.00 – 651.50)	71.00 (21.00 – 107.50)	47	494.00 (420.00 – 576.00)	589.00 (504.00 – 641.00)	76.00 (25.00 – 119.00)	0.462	0.644	0.045
Gait velocity [m/s]	61	1.49 (1.36 – 1.65)	1.72 (1.51 – 1.91)	0.22 (0.05 – 0.40)	47	1.47 (1.24 – 1.71)	1.65 (1.51 – 1.83)	0.18 (0.03 – 0.38)	-0.641	0.521	-0.062
$Watt_{max}$ [W]	31	90.00 (73.00 – 113.00)	108.00 (83.00 - 130.00)	16.00 (-4.00 – 30.00)	19	104.00 (93.00 – 112.00)	112.00 (89.00 – 146.00)	13.00 (-6.00 – 30.00)	-0.420	0.675	-0.059
$VO2_{max}$ [l/Min]	31	1.34 (1.18 – 1.53)	1.45 (1.31 – 1.68)	0.16 (0.00 – 0.37)	19	1.48 (1.43 – 1.81)	1.58 (1.38 – 1.89)	0.13 (-0.07 – 0.30)	-0.780	0.436	-0.110
1MSTST [n/Min]	60	19.50 (16.00 – 23.00)	20.50 (17.00 – 25.00)	2.00 (-1.75 – 4.75)	47	21.00 (16.00 – 25.00)	21.00 (17.00 – 28.00)	0.00 (-1.00 – 4.00)	-0.492	0.623	-0.048
Quadriceps strength [kg]	61	93.43 (68.85 – 133.09)	106.71 (86.10 – 139.68)	11.41 (-0.54 – 29.41)	47	99.05 (80.05 – 133.97)	108.35 (94.43 – 144.61)	10.71 (0.53 – 18.72)	-0.400	0.689	-0.039
Handgrip strength [kg]	61	27.10 (20.47 – 34.55)	26.77 (22.08 – 33.12)	-0.10 (-2.92 – 4.22)	47	29.80 (21.57 – 35.50)	29.53 (24.43 – 36.97)	1.27 (-3.33 – 3.77)	0.415	0.678	0.040
Balance function [0-36 points]	61	25.00 (20.00 – 27.00)	26.00 (22.00 – 30.00)	2.00 (0.00 – 5.00)	47	26.00 (22.00 – 28.00)	27.00 (22.00 – 28.00)	1.00 (-4.00 – 4.00)	-1.612	0.107	-0.155
Subjective physical ability [0-10 points]	65	4.89 (3.44 – 6.00)	5.56 (3.89 – 6.67)	0.56 (-0.28 – 1.61)	52	4.61 (3.36 – 6.06)	4.94 (3.39 – 6.22)	0.28 (-0.42 – 1.42)	-0.609	0.542	-0.056
PCFS [grade 0-4]	65	3.00 (2.00 – 3.00)	3.00 (2.00 – 3.00)	0.00 (0.00 – 0.00)	52	3.00 (2.00 – 3.00)	3.00 (2.00 – 3.00)	0.00 (0.00 – 0.00)	0.041	0.967	0.004
Subjective post-COVID symptoms [0-10 points]	65	3.00 (2.00 – 6.00)	3.00 (2.00 - 5.00)	0.00 (-2.00 – 2.00)	52	2.00 (1.25 – 6.00)	4.00 (2.00 – 6.00)	0.00 (-1.00 – 3.00)	1.233	0.218	0.114

IQR = interquartile range.

Table S12: Groupwise comparison of physical health of patients with pre-existing respiratory disease and without a pre-existing respiratory disease between timepoints T1 and T4.

	No				Yes				Between-group difference		
	N	T1 Median (IQR)	T4 Median (IQR)	Δ	N	T1 Median (IQR)	T4 Median (IQR)	Δ	z	p	r
6MWD [m]	56	520.50 (471.75 – 569.75)	568.00 (515.75 – 623.50)	44.00 (3.00 – 83.25)	39	522.00 (432.00 – 583.00)	571.00 (482.00 – 637.00)	49.00 (28.00 – 88.00)	0.828	0.407	0.085
Gait velocity [m/s]	56	1.49 (1.38 – 1.68)	1.64 (1.45 – 1.83)	0.14 (0.00 – 0.30)	39	1.44 (1.24 – 1.71)	1.67 (1.47 – 1.84)	0.17 (0.03 – 0.32)	0.643	0.520	0.066
$Watt_{max}$ [W]	31	92.00 (73.00 – 113.00)	108.00 (84.00 – 130.00)	15.00 (5.00 – 28.00)	20	105.00 (93.50 – 124.75)	111.50 (84.25 – 147.50)	10.00 (-10.25 – 29.75)	-0.540	0.589	-0.076
$VO2_{max}$ [l/Min]	31	1.30 (1.17 – 1.53)	1.43 (1.20 – 1.79)	0.13 (-0.00 – 0.38)	20	1.48 (1.42 – 1.82)	1.53 (1.31 – 1.96)	0.04 (-0.03 – 0.28)	-0.965	0.335	-0.135
1MSTST [n/Min]	55	19.00 (16.00 – 23.00)	20.00 (17.00 – 27.00)	2.00 (-1.00 – 5.00)	41	20.00 (16.00 – 24.00)	21.00 (17.00 – 25.50)	2.00 (-1.50 – 4.00)	-0.899	0.369	-0.092
Quadriceps strength [kg]	55	93.43 (6850 – 135.67)	111.91 (80.39 – 158.93)	19.05 (0.24 – 36.61)	41	99.05 (82.09 – 133.05)	116.89 (90.33 – 159.31)	16.96 (1.89 – 41.77)	0.233	0.816	0.024
Handgrip strength [kg]	56	26.15 (19.98 – 33.98)	25.80 (19.35 – 34.45)	-0.28 (-5.91 – 3.73)	42	30.25 (21.08 – 36.71)	31.82 (25.48 – 38.19)	0.52 (-3.02 – 4.03)	0.958	0.338	0.097
Balance function [0-36 points]	56	25.00 (20.50 – 27.00)	26.00 (21-25 – 29.75)	1.00 (-2.00 – 5.00)	40	25.00 (22.00 – 28.00)	26.00 (23.00 – 29.00)	1.00 (0.00 – 3.00)	0.280	0.780	0.029
Subjective physical ability [0-10 points]	63	4.89 (3.44 – 6.11)	5.11 (4.11 – 6.56)	0.44 (-0.56 – 1.44)	49	4.67 (3.50 – 6.22)	5.56 (3.89 – 6.56)	0.67 (-0.39 – 1.56)	0.355	0.723	0.036
PCFS [grade 0-4]	63	2.00 (2.00 – 3.00)	3.00 (2.00 – 3.00)	0.00 (0.00 – 0.00)	49	3.00 (2.00 – 3.00)	3.00 (1.50 – 3.00)	0.00 (-1.00 – 0.00)	-0.455	0.649	-0.043
Subjective post-COVID symptoms [0-10 points]	63	3.00 (2.00 – 6.00)	3.00 (2.00 – 6.00)	0.00 (-1.00 – 1.00)	49	2.00 (2.00 – 6.00)	5.00 (2.00 – 8.00)	1.00 (-1.00 – 4.00)	1.585	0.113	0.149

IQR = interquartile range.

Table S13: Groupwise comparison of physical health of patients with pre-existing metabolic disease and without a pre-existing metabolic disease between timepoints T1 and T3.

	No				Yes				Between-group difference		
	N	T1 Median (IQR)	T3 Median (IQR)	Δ	N	T1 Median (IQR)	T3 Median (IQR)	Δ	z	p	r
6MWD [m]	38	573.00 (507.75 – 630.25)	640.50 (597.25 – 680.00)	67.00 (21.00 – 110.50)	70	493.50 (420.75 – 544.50)	566.50 (502.75 – 612.75)	76.00 (22.00 – 116.75)	0.347	0.728	0.033
Gait velocity [m/s]	38	1.64 (1.43 – 1.77)	1.82 (1.65 – 2.00)	0.20 (0.04 – 0.33)	70	1.43 (1.23 – 1.59)	1.62 (1.45 – 1.81)	0.21 (0.04 – 0.41)	0.602	0.547	0.058
$Watt_{max}$ [W]	18	108.50 (92.00 – 133.00)	119.50 (104.75 – 154.75)	17.50 (-0.25 – 28.75)	32	89.50 (72.00 – 107.00)	102.50 (80.75 – 123.75)	15.00 (-4.75 – 30.00)	-0.071	0.944	-0.010
$VO2_{max}$ [l/Min]	18	1.44 (1.30 – 1.79)	1.60 (1.41 – 1.91)	0.20 (0.01 – 0.39)	32	1.43 (1.17 – 1.55)	1.44 (1.31 – 1.76)	0.10 (-0.03 – 0.26)	-0.990	0.322	-0.140
1MSTST [n/Min]	37	21.00 (18.50 – 25.00)	25.00 (19.00 – 31.00)	1.00 (-1.00 – 7.00)	70	19.00 (15.75 – 23.00)	19.50 (16.00 – 24.00)	1.00 (-2.00 – 4.00)	-0.684	0.494	-0.066
Quadriceps strength [kg]	38	95.71 (82.35 – 137.23)	119.06 (92.84 – 140.39)	10.44 (-3.55 – 20.49)	70	97.85 (69.14 – 126.28)	108.11 (86.25 – 143.29)	11.26 (0.31 – 34.18)	1.049	0.294	0.101
Handgrip strength [kg]	38	31.32 (23.93 – 36.81)	29.17 (21.73 – 36.22)	-1.07 (-3.43 – 1.93)	70	26.62 (20.17 – 33.28)	29.02 (23.12 – 33.40)	1.48 (-2.78 – 4.70)	2.014	0.044	0.194
Balance function [0-36 points]	38	26.00 (22.75 – 30.00)	28.00 (23.75 – 31.25)	1.50 (-1.00 – 5.00)	70	25.00 (20.00 – 27.00)	26.00 (21.75 – 29.00)	2.00 (-2.00 – 5.00)	0.094	0.926	0.009
Subjective	40	4.72 (3.81 – 6.17)	5.56 (4.14 – 7.28)	0.78 (0.00 – 1.64)	77	4.67 (3.39 – 5.83)	5.00 (3.61 – 6.17)	0.22 (-0.78 – 1.50)	-1.308	0.191	-0.121

Table S13: Groupwise comparison of physical health of patients with pre-existing metabolic disease and without a pre-existing metabolic disease between timepoints T1 and T3.

	No				Yes				Between-group difference		
	**N**	**T1 Median (IQR)**	**T3 Median (IQR)**	**Δ**	**N**	**T1 Median (IQR)**	**T3 Median (IQR)**	**Δ**	**z**	**p**	**r**
physical ability [0-10 points]											
PCFS [grade 0-4]	40	3.00 (2.00 – 3.00)	3.00 (1.25 – 3.00)	0.00 (-0.75 – 0.00)	77	3.00 (2.00 – 3.00)	3.00 (2.00 – 3.00)	0.00 (0.00 – 0.00)	1.406	0.160	0.130
Subjective post-COVID symptoms [0-10 points]	40	3.00 (1.00 – 5.00)	3.00 (2.00 – 6.75)	1.00 (-1.00 – 3.00)	77	3.00 (2.00 – 6.50)	3.00 (2.00 – 5.50)	0.00 (-2.00 – 2.00)	-2.120	0.034	-0.196

IQR = interquartile range.

Table S14: Groupwise comparison of physical health of patients with pre-existing metabolic disease and without a pre-existing metabolic disease between timepoints T1 and T4.

	No				Yes				Between-group difference		
	**N**	**T1 Median (IQR)**	**T4 Median (IQR)**	**Δ**	**N**	**T1 Median (IQR)**	**T4 Median (IQR)**	**Δ**	**z**	**p**	**r**
6MWD [m]	35	582.00 (498.00 – 631.00)	618.00 (567.00 – 698.00)	45.00 (17.00 – 107.00)	60	494.50 (432.00 – 543.00)	539.50 (486.00 – 597.50)	47.00 (7.75 – 83.25)	-0.366	0.714	-0.038
Gait velocity [m/s]	35	1.64 (1.43 – 1.78)	1.81 (1.61 – 1.93)	0.14 (-0.35 – 0.38)	60	1.43 (1.23 – 1.57)	1.61 (1.44 – 1.72)	0.17 (0.04 – 0.30)	0.532	0.594	0.057
$Watt_{max}$ [W]	21	108.00 (92.00 – 124.50)	120.00 (83.00 – 144.50)	14.00 (8.50 – 29.00)	30	91.00 (71.50 – 109.75)	100.50 (84.00 – 132.25)	13.50 (1.75 – 28.50)	0.392	0.695	0.055
$VO2_{max}$ [l/Min]	21	1.39 (1.23 – 1.67)	1.60 (1.30 – 1.91)	0.18 (-0.03 – 0.39)	30	1.43 (1.16 - 1.53)	1.48 (1.20 – 1.79)	0.09 (0.00 – 0.26)	-0.230	0.818	-0.032
1MSTST [n/Min]	35	22.00 (19.00 – 25.00)	25.00 (19.00 – 34.00)	3.00 (-1.00 – 6.00)	61	19.00 (15.00 – 22.00)	20.00 (-1.00 – 4.00)	2.00 (-1.00 – 4.00)	-1.672	0.095	-0.171
Quadriceps strength [kg]	35	98.46 (82.62 – 137.91)	116.05 (88.76 – 157.40)	17.52 (0.80 – 36.61)	61	98.60 (67.52 – 127.60)	115.52 (84.89 – 162.90)	21.13 (1.27 – 39.86)	0.666	0.505	0.068
Handgrip strength [kg]	36	31.32 (23.51 – 37.23)	30.80 (20.63 – 37.63)	-1.07 (-5.03 – 2.90)	62	26.03 (19.72 – 33.53)	27.98 (21.48 – 34.74)	0.52 (-3.77 – 4.71)	1.374	0.169	0.139
Balance function [0-36 points]	35	26.00 (23.00 – 30.00)	28.00 (24.00 – 30.00)	1.00 (-1.00 – 4.00)	61	24.00 (20.00 – 27.00)	25.00 (22.50 – 27.00)	1.00 (-1.50 – 3.00)	-0.623	0.534	-0.064
Subjective physical ability [0-10 points]	41	4.67 (3.72 – 6.11)	5.67 (3.78 – 7.28)	0.67 (-0.28 – 1.67)	71	4.78 (3.44 – 6.11)	5.00 (4.00 – 6.44)	0.33 (-0.56 – 1.22)	-1.190	0.234	-0.112
PCFS [grade 0-4]	41	3.00 (2.00 – 3.00)	2.00 (1.50 – 3.00)	0.00 (-0.50 – 0.00)	71	3.00 (2.00 – 3.00)	3.00 (2.00 – 3.00)	0.00 (0.00 – 0.00)	1.208	0.227	0.114
Subjective post-COVID symptoms [0-10 points]	41	3.00 (1.00 – 5.00)	3.00 (2.00 – 6.50)	0.00 (-1.50 – 2.00)	71	3.00 (2.00 – 6.00)	5.00 (2.00 – 7.00)	0.00 (-1.00 – 2.00)	0.228	0.819	0.022

IQR = interquartile range.

Table S15: Groupwise comparison of physical health of patients with pre-existing psychological disease and without a pre-existing psychological disease timepoints T1 and T3.

	No				Yes				Between-group difference		
	N	T1 Median (IQR)	T3 Median (IQR)	Δ	N	T1 Median (IQR)	T3 Median (IQR)	Δ	z	p	r
6MWD [m]	89	524.00 (457.50 – 583.00)	600.00 (519.00 – 662.00)	71.00 (19.50 – 118.50)	19	510.00 (391.00 – 534.00)	577.00 (504.00 – 609.00)	91.00 (56.00 – 108.00)	1.537	0.124	0.148
Gait velocity [m/s]	89	1.51 (1.35 – 1.70)	1.69 (1.53 – 1.93)	0.18 (0.02 – 0.38)	19	1.39 (1.14 – 1.52)	1.65 (1.51 – 1.83)	0.31 (0.12 – 0.41)	1.537	0.124	0.148
$Watt_{max}$ [W]	41	97.00 (75.50 – 115.50)	111.00 (91.00 – 141.50)	16.00 (-2.00 – 30.50)	9	88.00 (75.00 – 106.50)	103.00 (76.00 – 108.00)	-4.0 (-7.00 – 27.00)	0.256	0.261	0.036
$VO2_{max}$ [l/Min]	41	1.43 (1.23 – 1.57)	1.53 (1.36 – 1.85)	0.16 (-0.01 – 0.36)	9	1.47 (1.16 – 1.54)	1.42 (1.19 – 1.54)	0.01 (-0.11 – 0.28)	0.240	0.250	0.034
1MSTST [n/Min]	89	21.00 (16.50 – 24.00)	21.00 (17.50 – 25.00)	1.00 (-2.00 – 4.00)	18	18.00 (15.75 – 23.50)	20.50 (15.00 – 26.25)	1.00 (-0.25 – 5.00)	0.544	0.587	0.053
Quadriceps strength [kg]	89	95.43 (73.89 – 135.99)	108.11 (92.13 – 143.66)	10.88 (0.08 – 27.68)	19	105.61 (74.28 – 119.46)	116.12 (86.56 – 127.50)	11.41 (-0.70 – 31.97)	0.311	0.756	0.030
Handgrip strength [kg]	89	29.27 (20.73 – 35.18)	29.13 (22.90 – 36.47)	0.83 (-2.67 – 4.22)	19	26.17 (23.30 – 33.70)	29.53 (20.67 – 32.07)	-0.27 (-6.43 – 3.70)	-0.783	0.434	-0.075
Balance function [0-36 points]	89	26.00 (22.00 – 27.50)	27.00 (23.00 – 30.00)	1.00 (-2.00 – 5.00)	19	20.00 (15.00 – 26.00)	22.00 (15.00 – 30.00)	2.00 (1.00 - 7.00)	1.298	0.194	0.125
Subjective physical ability [0-10 points]	95	4.78 (3.56 – 6.22)	5.00 (3.78 – 6.44)	0.22 (-0.67 – 1.44)	22	4.22 (2.56 – 4.89)	5.67 (3.94 – 6.56)	1.22 (0.00 – 1.75)	2.122	0.034	0.196
PCFS [grade 0-4]	95	3.00 (2.00 – 3.00)	3.00 (2.00 – 3.00)	0.00 (0.00 – 0.00)	22	3.00 (2.00 – 3.00)	2.50 (2.00 – 3.00)	0.00 (-0.25 – 0.00)	-0.727	0.467	-0.067
Subjective post-COVID symptoms [0-10 points]	95	3.00 (2.00 – 6.00)	3.00 (2.00 - 6.00)	0.00 (-2.00 – 2.00)	22	2.00 (1.00 – 5.25)	3.00 (2.00 – 4.50)	1.00 (-2.00 – 2.00)	0.386	0.700	0.036

IQR = interquartile range.

Table S16: Groupwise comparison of physical health of patients with pre-existing psychological disease and without a pre-existing psychological disease between timepoints T1 and T4.

	No				Yes				Between-group difference		
	N	T1 Median (IQR)	T4 Median (IQR)	Δ	N	T1 Median (IQR)	T4 Median (IQR)	Δ	z	p	r
6MWD [m]	79	524.00 (460.00 – 585.00)	580.00 (509.00 – 640.00)	45.00 (6.00 – 85.00)	16	513.50 (392.50 – 532.25)	548.00 (488.00 – 584.50)	57.50 (23.00 – 102.00)	0.617	0.538	0.063
Gait velocity [m/s]	79	1.49 (1.36 – 1.70)	1.67 (1.50 – 1.84)	0.16 (0.02 – 0.30)	16	1.41 (1.16 – 1.57)	1.58 (1.36 – 1.69)	0.14 (0.04 – 0.40)	0.194	0.846	0.020
$Watt_{max}$ [W]	41	104.00 (75.50 – 116.50)	118.00 (84.50 – 143.50)	15.00 (1.50 – 29.00)	10	86.50 (76.00 – 103.75)	100.50 (74.25 – 120.25)	10.00 (-17.00 – 24.50)	-0.783	0.434	-0.110
$VO2_{max}$ [l/Min]	41	1.41 (1.22 – 1.57)	1.51 (1.33 – 1.82)	0.12 (0.00 – 0.37)	10	1.38 (1.17 – 1.53)	1.49 (1.18 – 1.66)	0.05 (-0.10 – 0.29)	-0.569	0.569	-0.080
1MSTST [n/Min]	80	20.00 (16.25 – 24.00)	21.00 (17.00 – 26.75)	2.00 (-1.00 – 5.00)	16	17.00 (15.25 – 22.50)	20.00 (16.50 – 24.00)	2.00 (0.00 – 5.75)	0.286	0.775	0.029
Quadriceps strength [kg]	80	96.95 (74.23 – 136.13)	113.98 (86.79 – 158.79)	19.50 (0.97 – 36.47)	16	105.64 (70.47 – 123.01)	118.50 (90.65 – 159.37)	16.78 (1.67 – 44.51)	0.619	0.536	0.063
Handgrip strength [kg]	81	28.63 (20.22 – 35.50)	28.00 (20.87 – 36.73)	-0.07 (-4.87 – 3.23)	17	25.90 (22.63 – 32.43)	30.80 (23.47 – 34.47)	2.53 (-3.82 – 5.83)	0.966	0.334	0.098
Balance function [0-36 points]	79	25.00 (22.00 – 28.00)	26.00 (23.00 – 30.00)	1.00 (-1.00 – 3.00)	17	20.00 (17.50 – 25.50)	22.00 (17.00 – 28.00)	2.00 (-1.00 – 3.50)	0.655	0.513	0.067
Subjective physical ability [0-10 points]	91	4.89 (3.67 – 6.22)	5.33 (4.11 – 6.56)	0.56 (-0.56 – 1.56)	21	4.22 (2.67 – 4.89)	5.00 (3.78 – 6.39)	0.78 (0.06 – 1.50)	1.093	0.275	0.103

Table S16: Groupwise comparison of physical health of patients with pre-existing psychological disease and without a pre-existing psychological disease between timepoints T1 and T4.

	No				Yes				Between-group difference		
	N	T1 Median (IQR)	T4 Median (IQR)	Δ	N	T1 Median (IQR)	T4 Median (IQR)	Δ	z	p	r
PCFS [grade 0-4]	91	3.00 (2.00 – 3.00)	2.00 (0.00 – 3.00)	0.00 (-1.00 – 0.00)	21	3.00 (2.00 – 3.00)	3.00 (2.00 – 3.00)	0.00 (0.00 – 0.00)	0.683	0.495	0.065
Subjective post-COVID symptoms [0-10 points]	91	3.00 (2.00 – 6.00)	4.00 (2.00 – 7.00)	0.00 (-1.00 – 2.00)	21	2.00 (2.00 – 5.50)	3.00 (1.50 – 6.00)	0.00 (-2.00 – 2.00)	-0.628	0.530	-0.059

IQR = interquartile range.

Table S17: Groupwise comparison of physical health of patients with pre-existing neuro-sensory disease and without a pre-existing neuro-sensory disease between timepoints T1 and T3.

	No				Yes				Between-group difference		
	N	T1 Median (IQR)	T3 Median (IQR)	Δ	N	T1 Median (IQR)	T3 Median (IQR)	Δ	z	p	r
6MWD [m]	72	519.50 (448.50 – 575.25)	600.50 (546.25 – 639.75)	70.00 (25.00 – 118.25)	36	510.00 (437.75 – 575.75)	563.00 (453.25 – 676.25)	76.00 (14.50 – 115.25)	-0.489	0.625	-0.047
Gait velocity [m/s]	72	1.48 (1.35 – 1.65)	1.71 (1.54 – 1.87)	0.23 (0.09 – 0.39)	36	1.47 (1.27 – 1.71)	1.65 (1.38 – 2.00)	0.17 (0.00 – 0.37)	-1.131	0.258	-0.109
$Watt_{max}$ [W]	30	105.00 (77.00 – 121.25)	110.00 (96.75 – 149.50)	17.50 (-1.75 – 32.75)	20	90.50 (70.50 – 101.75)	99.00 (80.75 – 123.25)	14.00 (-5.50 – 26.50)	-1.109	0.267	-0.157
$VO2_{max}$ [l/Min]	30	1.45 (1.23 – 1.71)	1.57 (1.35 – 1.90)	0.16 (0.02 – 0.37)	20	1.39 (1.19 - 1.49)	1.43 (1.28 – 1.62)	0.02 (-0.05 – 0.30)	-1.248	0.212	-0.177
1MSTST [n/Min]	72	20.00 (17.00 – 24.00)	22.00 (18.00 – 26.00)	2.00 (-1.75 – 5.00)	35	20.00 (16.00 – 24.00)	19.00 (16.00 – 23.00)	0.00 (-1.00 – 3.00)	-1.284	0.199	-0.124
Quadriceps strength [kg]	72	105.11 (79.27 – 138.10)	122.23 (94.53 – 151.40)	13.85 (2.58 – 30.28)	36	92.41 (66.27 – 121.92)	101.54 (81.22 – 126.06)	5.81 (-3.58 – 18.02)	-1.564	0.118	-0.151
Handgrip strength [kg]	72	28.67 (20.66 – 35.50)	29.57 (22.88 – 35.90)	1.51 (-1.87 – 4.63)	36	25.13 (22.22 – 34.80)	26.82 (22.29 – 32.24)	-1.38 (-5.13 – 2.58)	-2.177	0.029	-0.210
Balance function [0-36 points]	72	25.00 (22.00 – 27.00)	27.50 (23.25 – 30.00)	2.00 (-0.75 – 5.00)	36	25.00 (20.00 – 27.00)	25.50 (18.25 – 28.00)	0.50 (-4.00 – 5.75)	-1.476	0.140	-0.142
Subjective physical ability [0-10 points]	77	4.89 (3.50 – 6.11)	5.33 (3.72 – 6.56)	0.33 (-0.39 – 1.39)	40	4.50 (3.28 – 5.44)	4.94 (3.92 – 6.33)	0.83 (-0.28 – 1.56)	0.584	0.560	0.054
PCFS [grade 0-4]	77	3.00 (2.00 – 3.00)	3.00 (2.00 – 3.00)	0.00 (0.00 – 0.00)	40	2.00 (2.00 – 3.00)	3.00 (2.00 – 3.00)	0.00 (0.00 – 1.00)	1.528	0.127	0.141
Subjective post-COVID symptoms [0-10 points]	77	2.00 (1.00 – 6.00)	3.00 (2.00 – 6.00)	0.00 (-1.50 – 2.00)	40	3.00 (2.00 – 6.75)	3.00 (2.00 – 6.00)	0.00 (-2.00 – 2.00)	-0.607	0.544	-0.056

IQR = interquartile range.

Table S18: Groupwise comparison of physical health of patients with pre-existing neuro-sensory disease and without a pre-existing neuro-sensory disease between timepoints T1 and T4.

	No				Yes				Between-group difference		
	**N**	**T1 Median (IQR)**	**T4 Median (IQR)**	**Δ**	**N**	**T1 Median (IQR)**	**T4 Median (IQR)**	**Δ**	**z**	**p**	**r**
6MWD [m]	67	520.00 (448.00 – 581.00)	580.00 (509.00 – 619.00)	44.00 (17.00 – 84.00)	28	523.00 (472.50 – 575.75)	539.00 (494.00 – 640.00)	49.00 (2.50 – 104.00)	0.188	0.851	0.019
Gait velocity [m/s]	67	1.47 (1.35 – 1.64)	1.66 (1.47 – 1.83)	0.17 (0.05 – 0.32)	28	1.47 (1.32 – 1.72)	1.64 (1.48 – 1.83)	0.11 (-0.12 – 0.32)	-1.131	0.258	-0.116
Watt$_{max}$ [W]	33	104.00 (77.00 – 118.50)	114.00 (84.50 – 147.00)	14.00 (-1.00 – 29.00)	18	93.50 (71.50 – 108.00)	100.00 (79.50 – 132.25)	13.50 (0.25 – 29.25)	0.069	0.945	0.010
VO2$_{max}$ [l/Min]	33	1.41 (1.20 – 1.63)	1.50 (1.31 – 1.84)	0.11 (-0.03 – 0.33)	18	1.38 (1.20 – 1.48)	1.47 (1.18 – 1.82)	0.13 (-0.00 – 0.38)	0.306	0.760	0.043
1MSTST [n/Min]	67	20.00 (16.00 – 23.00)	21.00 (18.00 – 26.00)	2.00 (-1.00 – 5.00)	29	20.00 (16.00 – 24.00)	20.00 (17.00 – 29.00)	2.00 (-2.00 – 5.50)	-0.308	0.758	-0.031
Quadriceps strength [kg]	68	96.95 (75.84 – 136.72)	116.72 (87.59 – 171.58)	21.24 (3.22 – 38.05)	28	98.83 (67.88 – 123.85)	112.45 (82.17 – 131.14)	12.12 (-1.30 – 39.70)	-1.056	0.291	-0.108
Handgrip strength [kg]	68	27.77 (20.13 – 35.75)	29.45 (21.59 – 36.81)	0.47 (-4.24 – 3.88)	30	24.78 (21.87 – 34.42)	28.33 (20.30 – 35.03)	-0.52 (-5.06 – 3.88)	-0.382	0.703	-0.039
Balance function [0-36 points]	67	25.00 (22.00 – 27.00)	26.00 (23.00 – 29.00)	1.00 (-1.00 – 3.00)	29	25.00 (20.00 – 28.00)	26.00 (21.50 – 30.00)	2.00 (-1.50 – 4.50)	0.496	0.620	0.051
Subjective physical ability [0-10 points]	74	5.00 (3.75 – 6.22)	5.39 (4.31 – 6.58)	0.56 (-0.56 – 1.47)	38	4.50 (3.14 – 5.61)	5.06 (3.22 – 6.47)	0.67 (-0.33 – 1.72)	0.332	0.740	0.031
PCFS [grade 0-4]	74	3.00 (2.00 – 3.00)	2.50 (0.00 – 3.00)	0.00 (-1.00 – 0.00)	38	2.00 (2.00 – 3.00)	3.00 (2.00 – 3.00)	0.00 (0.00 – 0.00)	0.798	0.425	0.075
Subjective post-COVID symptoms [0-10 points]	74	3.00 (1.75 – 6.00)	3.00 (2.00 – 6.25)	0.00 (-1.00 – 2.009	38	3.00 (2.00 – 6.25)	5.00 (2.75 – 7.25)	1.00 (-1.00 – 3.00)	1.190	0.234	0.112

IQR = interquartile range.

Table S19: Groupwise comparison of physical health of patients with pre-existing musculoskeletal disease and without a pre-existing musculoskeletal disease between timepoints T1 and T3.

	No				Yes				Between-group difference		
	**N**	**T1 Median (IQR)**	**T3 Median (IQR)**	**Δ**	**N**	**T1 Median (IQR)**	**T3 Median (IQR)**	**Δ**	**z**	**p**	**r**
6MWD [m]	38	515.50 (420.75 – 578.00)	605.00 (536.25 – 661.50)	76.50 (20.25 – 120.25)	70	520.50 (458.75 – 577.25)	581.50 (517.00 – 639.25)	71.50 (22.00 – 113.75)	-0.466	0.641	-0.045
Gait velocity [m/s]	38	1.44 (1.25 – 1.66)	1.67 (1.43 – 1.97	0.19 (0.06 – 0.42)	70	1.50 (1.35 – 1.67)	1.69 (1.52 – 1.87)	0.21 (0.03 – 0.38)	-0.135	0.893	-0.013
Watt$_{max}$ [W]	22	91.00 (71.50 – 112.25)	107.50 (82.25 – 127.25)	16.50 (-4.25 – 31.25)	28	101.00 (82.00 – 117.50)	108.50 (93.50 – 143.50)	15.50 (-3.75 – 27.75)	-0.479	0.632	-0.068
VO2$_{max}$ [l/Min]	22	1.33 (1.13 – 1.56)	1.47 (1.31 – 1.81)	0.14 (0.03 – 0.37)	28	1.46 (1.25 – 1.72)	1.52 (1.38 – 1.86)	0.14 (-0.05 – 0.29)	-1.134	0.257	-0.160
1MSTST [n/Min]	38	20.00 (15.75 – 23.00)	20.50 (16.75 – 25.50)	2.00 (-1.00 – 7.00)	69	20.00 (16.00 – 25.00)	21.00 (17.00 – 26.00)	0.00 (-2.00 – 4.00)	-1.559	0.119	-0.151
Quadriceps strength [kg]	38	93.19 (77.60 – 136.42)	123.63 (95.51 – 143.26)	14.92 (0.59 – 35.94)	70	99.79 (73.21 – 132.42)	108.23 (88.10 – 140.39)	10.68 (-0.45 – 25.11)	-0.630	0.528	-0.061
Handgrip strength [kg]	38	27.77 (22.24 – 35.25)	30.18 (23.24 – 35.52)	0.45 (-2.63 – 2.66)	70	28.57 (20.78 – 35.10)	28.03 (22.53 – 34.53)	1.03 (-3.83 – 4.28)	0.048	0.962	0.005
Balance function [0-36 points]	38	26.00 (22.00 – 28.25)	28.00 (23.50 – 30.25)	2.50 (0.00 – 5.00)	70	25.00 (20.75 – 27.00)	26.00 (21.75 – 29.00)	1.00 (-3.00 – 5.00)	-0.922	0.356	-0.089
Subjective physical ability [0-10 points]	40	4.72 (2.89 – 6.86)	5.11 (3.81 – 6.36)	0.17 (-0.33 – 1.47)	77	4.67 (3.67 – 5.78)	5.22 (3.78 – 6.50)	0.56 (-0.33 – 1.56)	0.520	0.603	0.048

Table S19: Groupwise comparison of physical health of patients with pre-existing musculoskeletal disease and without a pre-existing musculoskeletal disease between timepoints T1 and T3.

	No				Yes				Between-group difference		
	**N**	**T1 Median (IQR)**	**T3 Median (IQR)**	**Δ**	**N**	**T1 Median (IQR)**	**T3 Median (IQR)**	**Δ**	**z**	**p**	**r**
PCFS [grade 0-4]	40	3.00 (2.00 – 3.00)	3.00 (2.00 – 3.00)	0.00 (0.00 – 0.00)	77	3.00 (2.00 – 3.00)	3.00 (2.00 – 3.00)	0.00 (0.00 – 0.00)	-0.319	0.749	-0.030
Subjective post-COVID symptoms [0-10 points]	40	3.00 (1.00 – 5.00)	4.00 (3.00 – 6.00)	1.00 (-0.75 – 3.00)	77	3.00 (2.00 – 6.00)	3.00 (2.00 – 6.00)	0.00 (-2.00 – 2.00)	-2.401	0.016	-0.222

IQR = interquartile range.

Table S20: Groupwise comparison of physical health of patients with pre-existing musculoskeletal disease and without a pre-existing musculoskeletal disease between timepoints T1 and T4.

	No				Yes				Between-group difference		
	**N**	**T1 Median (IQR)**	**T4 Median (IQR)**	**Δ**	**N**	**T1 Median (IQR)**	**T4 Median (IQR)**	**Δ**	**z**	**p**	**r**
6MWD [m]	33	511.00 (420.50 – 586.00)	562.00 (487.50 – 629.00)	40.00 (16.00 – 82.50)	62	521.50 (470.50 – 581.25)	575.50 (507.75 – 630.50)	48.50 (15.25 – 88.25)	0.590	0.555	0.061
Gait velocity [m/s]	33	1.44 (1.33 – 1.67)	1.64 (1.42 – 1.93)	0.13 (-0.02 – 0.35)	62	1.49 (1.34 – 1.68)	1.66 (1.51 – 1.82)	0.17 (0.04 – 0.31)	0.066	0.947	0.007
$Watt_{max}$ [W]	18	92.50 (69.75 – 114.25)	106.50 (81.75 – 137.75)	13.50 (4.25 – 31.25)	33	101.00 (82.50 – 114.00)	108.00 (84.00 – 140.00)	14.00 (-7.00 – 28.50)	-0.384	0.701	-0.054
$VO2_{max}$ [l/Min]	18	1.31 (1.09 – 1.57)	1.48 (1.30 – 1.77)	0.18 (-0.02 – 0.41)	33	1.45 (1.23 – 1.53)	1.50 (1.24 – 1.85)	0.09 (-0.02 – 0.28)	-0.926	0.354	-0.130
1MSTST [n/Min]	35	20.00 (14.00 – 23.00)	21.00 (17.00 – 26.00)	3.00 (0.00 – 5.00)	61	20.00 (16.00 - 24.00)	21.00 (17.50 – 26.00)	1.00 (-1.00 – 5.00)	-1.172	0.241	-0.120
Quadriceps strength [kg]	34	92.59 (77.60 – 137.13)	118.51 (88.57 – 171.45)	20.10 (10.15 – 49.34)	62	99.79 (71.03 – 132.42)	113.98 (82.51 – 157.05)	17.96 (-0.83 – 36.78)	-1.279	0.201	-0.129
Handgrip strength [kg]	36	27.47 (21.79 – 35.47)	29.02 (20.78 – 37.26)	0.57 (-5.81 – 5.03)	62	27.80 (20.17 – 35.50)	28.75 (21.82 – 35.43)	-0.08 (-4.19 – 3.82)	-0.118	0.906	-0.012
Balance function [0-36 points]	34	26.00 (22.00 – 28.25)	26.00 (23.00 – 30.00)	1.00 (-3.25 – 3.50)	62	25.00 (20.00 – 27.00)	26.00 (22.75 – 29.00)	1.00 (-1.00 – 3.25)	0.484	0.628	0.049
Subjective physical ability [0-10 points]	39	4.89 (2.89 – 6.89)	5.33 (4.22 – 6.56)	0.44 (-0.33 – 1.22)	73	4.78 (3.72 – 5.94)	5.11 (3.89 – 6.61)	0.56 (-0.56 – 1.61)	0.406	0.684	0.038
PCFS [grade 0-4]	39	2.00 (2.00 – 3.00)	2.00 (0.00 – 3.00)	0.00 (0.00 – 0.00)	73	3.00 (2.00 – 3.00)	3.00 (2.00 – 3.00)	0.00 (-0.50 – 0.00)	0.323	0.747	0.031
Subjective post-COVID symptoms [0-10 points]	39	3.00 (2.00 – 5.00)	4.00 (2.00 – 6.00)	0.00 (-1.00 – 2.00)	73	3.00 (2.00 – 6.00)	4.00 (2.00 – 7.00)	0.00 (-1.00 – 2.00)	-0.530	0.596	-0.051

IQR = interquartile range.

Table S21: Groupwise comparison of physical health of patients with repeated rehabilitation and without repeated rehabilitation between timepoints T1 and T4.

	No				Yes				Between-group difference		
	N	T1 Median (IQR)	T4 Median (IQR)	Δ	N	T1 Median (IQR)	T4 Median (IQR)	Δ	z	p	r
6MWD [m]	70	523.00 (453.75 – 577.25)	569.00 (492.75 – 622.75)	46.00 (5.75 – 81.75)	22	520.50 (463.50 – 582.25)	594.50 (533.75 – 642.75)	45.00 (26.00 – 112.25)	-0.655	0.513	-0.068
Gait velocity [m/s]	70	1.50 (1.32 – 1.68)	1.65 (1.46 – 1.84)	0.17 (0.03 – 0.32)	22	1.45 (1.37 – 1.65)	1.67 (1.55 – 1.84)	0.15 (0.03 – 0.38)	-0.348	0.728	-0.036
$Watt_{max}$ [W]	40	105.00 (79.75 – 118.75)	121.50 (95.25 – 147.50)	16.50 (-1.25 – 29.75)	8	88. 00 (63.25 – 100.00)	79.50 (69.75 – 115.50)	13.50 (-20.50 – 23.00)	0.354	0.361	0.050
$VO2_{max}$ [l/Min]	40	1.42 (1.22 – 1.65)	1.55 (1.38 – 1.93)	0.15 (-0.07 – 0.37)	8	1.43 (0.98 – 1.47)	1.18 (0.84 – 1.74)	0.09 (-0.28 – 0.36)	1.037	0.300	0.145
1MSTST [n/Min]	70	20.00 (17.00 – 24.00)	21.00 (17.00 – 26.25)	2.00 (-1.00 – 4.00)	23	19.00 (15.00 – 21.00)	20.00 (17.00 – 26.00)	4.00 (0.00 – 6.00)	-1.996	0.046	-0.207
Quadriceps strength [kg]	71	104.61 (74.27 – 135.67)	116.55 (87.49 – 169.11)	21.02 (1.48 – 42.98)	22	83.19 (67.85 – 134.96)	112.61 (85.66 – 150.82)	18.67 (8.92 – 32.50)	0.298	0.765	0.031
Handgrip strength [kg]	72	28.67 (20.68 – 35.75)	28.92 (21.59 – 36.81)	-0.20 (-4.09 – 3.50)	23	25.90 (20.23 – 35.07)	29.03 (20.60 – 34.70)	1.80 (-5.13 – 6.33)	-0.956	0.339	-0.098
Balance function [0-36 points]	72	25.00 (22.00 – 27.00)	26.00 (23.00 – 29.00)	1.00 (-1.00 – 3.00)	21	23.00 (20.00 – 29.50)	26.00 (23.00 – 31.00)	2.00 (-2.00 – 5.00)	-0.848	0.396	-0.088
Subjective physical ability [0-10 points]	82	4.89 (3.83 – 6.22)	5.33 (4.22 – 6.58)	0.56 (-0.58 – 1.44)	28	4.11 (2.89 – 5.81)	5.39 (3.25 – 6.50)	0.50 (-0.08 – 1.83)	-1.057	0.290	-0.101
PCFS [grade 0-4]	83	2.00 (2.00 – 3.00)	2.00 (0.00 – 3.00)	0.00 (-1.00 – 0.00)	27	3.00 (2.00 – 3.00)	3.00 (2.00 – 3.00)	0.00 (0.00 – 0.00)	-1.119	0.263	-0.107
Subjective post-COVID symptoms [0-10 points]	82	4.00 (2.00 – 6.25)	3.50 (2.00 – 7.00)	0.00 (-1.00 – 2.00)	28	2.00 (1.00 - 3.00)	4.00 (2.00 – 6.75)	1.00 (-1.00 – 4.00)	-1.874	0.061	-0.179

IQR = interquartile range.

Table S22: Groupwise comparison of physical health of patients with exercising in an outpatient group and those not exercising in an outpatient group between timepoints T1 and T4.

	No				Yes				Between-group difference		
	N	T1 Median (IQR)	T4 Median (IQR)	Δ	N	T1 Median (IQR)	T4 Median (IQR)	Δ	z	p	r
6MWD [m]	71	525.00 (460.00 – 602.00)	583.00 (509.00 – 640.00)	46.00 (16.00 – 85.00)	16	518.50 (448.50 – 539.00)	562.00 (498.00 – 596.00)	46.50 (17.75 – 80.00)	0.153	0.878	0.016
Gait velocity [m/s]	71	1.49 (1.35 – 1.70)	1.69 (1.48 – 1.90)	0.17 (0.04 – 0.34)	16	1.45 (1.26 – 1.56)	1.60 (1.45 – 1.70)	0.14 (0.03 – 0.20)	1.030	0.303	0.110
$Watt_{max}$ [W]	36	107.00 (88.25 – 118.75)	108.50 (81.75 – 140.50)	7.00 (-12.25 – 24.50)	11	80.00 (65.00 – 104.00)	130.00 (87.00 – 148.00)	28.00 (17.00 – 64.00)	-2.853	0.004	-0.400
$VO2_{max}$ [l/Min]	36	1.44 (1.23 – 1.56)	1.48 (1.29 – 1.79)	0.08 (-0.63 – 0.22)	11	1.41 (1.02 – 1.58)	1.76 (1.35 – 1.96)	0.31 (0.09 – 0.39)	-2.324	0.020	-0.325
1MSTST [n/Min]	73	20.00 (16.00 – 24.00)	21.00 (17.00 – 27.00)	2.00 (-0.50 – 5.00)	15	17.00 (15.00 – 22.00)	20.00 (19.00 – 24.00)	3.00 (0.00 – 5.00)	-0.451	0.652	-0.048
Quadriceps strength [kg]	72	90.93 (74.43 – 134.20)	116.47 (93.24 – 166.56)	21.19 (4.55 – 40.55)	16	109.95 (68.13 – 150.14)	116.04 (79.63 – 158.06)	17.14 (-0.22 – 26.56)	1.309	0.191	0.140
Handgrip strength [kg]	74	28.07 (21.13 – 36.48)	29.57 (21.48 – 37.11)	-0.52 (-4.38 – 3.23)	16	27.98 (20.11 – 34.73)	28.42 (23.37 – 34.83)	1.28 (-5.25 – 5.48)	-0.945	0.345	-0.100
Balance function [0-36 points]	72	25.00 (22.00 – 27.75)	26.00 (23.00 – 29.75)	1.00 (-2.00 – 3.00)	16	22.50 (20.00 – 26.75)	25.00 (23.00 – 28.50)	1.00 (-0.75 – 4.75)	-0.705	0.481	-0.075
Subjective	87	4.89 (3.44 – 6.22)	5.33 (4.00 – 6.67)	0.56 (-0.56 – 1.56)	17	4.67 (4.17 – 5.17)	5.00 (3.50 – 6.22)	0.11 (-0.83 – 1.06)	1.091	0.275	0.108

Table S22: Groupwise comparison of physical health of patients with exercising in an outpatient group and those not exercising in an outpatient group between timepoints T1 and T4.

	No				Yes				Between-group difference		
	N	T1 Median (IQR)	T4 Median (IQR)	Δ	N	T1 Median (IQR)	T4 Median (IQR)	Δ	z	p	r
physical ability [0-10 points]											
PCFS [grade 0-4]	87	2.00 (2.00 – 3.00)	2.00 (2.00 – 3.00)	0.00 (0.00 – 0.00)	17	3.00 (2.00 – 3.00)	3.00 (1.00 – 3.00)	0.00 (-0.50 – 0.00)	-0.015	0.988	-0.002
Subjective post-COVID symptoms [0-10 points]	87	2.00 (1.00 – 5.00)	4.00 (2.00 – 7.00)	0.00 (-1.00 – 3.00)	17	5.00 (2.50 – 7.50)	4.00 (2.00 – 6.50)	-1.00 (-2.00 – 1.00)	2.102	0.036	0.207

IQR = interquartile range.
